# Allosteric Site Inhibitor Disrupting Auto-Processing of Malarial Cysteine Proteases

**DOI:** 10.1038/s41598-018-34564-8

**Published:** 2018-11-01

**Authors:** A. Pant, R. Kumar, N. A. Wani, S. Verma, R. Sharma, V. Pande, A. K. Saxena, R. Dixit, R. Rai, K. C. Pandey

**Affiliations:** 1 ICMR-National Institute of Malaria Research, Dwarka Sector 8, New Delhi, India; 2 Integrated Science Lab, Umeå University, Umeå, Sweden; 3 Medicinal Chemistry Division, CSIR-Indian Institute of Integrative Medicine, Jammu, India; 4 School of Life Sciences, Jawaharlal Nehru University, New Delhi, India; 5 Department of Biotechnology, Kumaun University, Nainital, Uttarakhand India; 6 Department of Biochemistry, ICMR-National Institute for Research in Environmental Health, Bhopal, MP - 462001 India

## Abstract

Falcipains are major haemoglobinases of *Plasmodium falciparum* required for parasite growth and development. They consist of pro- and mature domains that interact via ‘hot-spot’ interactions and maintain the structural integrity of enzyme in zymogen state. Upon sensing the acidic environment, these interactions dissociate and active enzyme is released. For inhibiting falcipains, several active site inhibitors exist, however, compounds that target via allosteric mechanism remains uncharacterized. Therefore, we designed and synthesized six azapeptide compounds, among which, NA-01 & NA-03 arrested parasite growth by specifically blocking the auto-processing of falcipains. Inhibitors showed high affinity for enzymes in presence of the prodomain without affecting the secondary structure. Binding of NA-03 at the interface induced rigidity in the prodomain preventing structural reorganization. We further reported a histidine-dependent activation of falcipain. Collectively, for the first time we provide a framework for blocking the allosteric site of crucial haemoglobinases of the human malaria parasite. Targeting the allosteric site could provide high selectivity and less vulnerable to drug resistance.

## Introduction

In the past fifteen years, malaria incidences among human population have reduced 21% globally, yet half of the world’s population still remains at risk^1^. *Plasmodium* parasite, the causative agent of malaria has a very complex life cycle that requires precise spatial and temporal regulation of numerous enzymes. Proteases, while only constitute ∼2% of the *Plasmodium* genome^2^, are involved in indispensable functions such as host haemoglobin (Hb) degradation, protein export, skeletal protein degradation, surface antigen processing etc^3–5^, thus could be considered as potential drug targets.

The genome of *P*. *falciparum* encodes cysteine proteases, falcipain-2 (FP2) and falcipain-3 (FP3), that have major roles in host Hb degradation which provide nutrients required for parasite survival^6,7^. FP2 and FP3 are predominantly expressed in trophozoite stage^8^. They are synthesized as zymogens of ∼50 kDa, that consist of bipartite pro- and mature domains which are further subdivided into inhibitory, refolding and Hb binding domains^4,9,10^. The inhibitory motifs ERFNIN and GNFD of prodomain cover the active site cleft of the mature domain and prevents falcipain activation^9,11,12^. The zymogens are transported through the endoplasmic reticulum (ER)/Golgi network to the food vacuole (FV), where under the influence of its acidic environment, the prodomain dissociates and releases ∼27 kDa active enzyme^13,14^. Earlier mutagenesis studies showed that certain residues that resides at the interface of pro- and mature domains are involved in the formation of salt bridges (R185-E221, E210-K403 in FP2 and R202-E238 in FP3) and hydrophobic interactions (F214, W449, W453 in FP2 and F231, W457, W461 in FP3)^15^. These interactions are essential for the dissociation of the prodomain and responsible for auto-processing, therefore considered as ‘hot-spot’ interactions.

Owing to the importance of falcipains during parasite growth and metabolism, various effective active site inhibitors such as E-64^16,17^, leupeptin^17,18^, vinyl sulfones^19–21^, peptidyl fluoromethyl ketones^22,23^ and falstatin^24,25^ (an endogenous macromolecular inhibitor) have been characterized. While recent advancement have extensively focused on blocking the active site of falcipains, studies describing compounds that inhibit allosterically, remain unexplored. Research associated with other diseases illustrated the possibility of targeting exosites in proteases by small molecule inhibitors/peptides, which could provide high potency and selectivity. In the human aspartic protease β-site of amyloid precursor protein cleaving enzyme (BACE), an inhibitor occupies the ligand binding site within the catalytic domain rather than the active site, which leads to concentration-dependent inhibition of substrate related to amyloid precursor protein (APP)^26^. Another study in human Kaposi’s sarcoma-associated herpesvirus (KSHV) showed that inhibitor DD2 binds at the interface of two monomers of KHSV serine proteases, stabilizes the zymogen-like conformation and prevents dimerization^27^. For recombinant human Cathepsin K, a compound NSC94914 was developed which binds at an allosteric pocket^28^. Recent studies in matrix metalloproteinases-9 (MMP-9) showed that JNJ0966 compound maintains the zymogen state of MMP-9 and inhibits the generation of catalytically active enzyme^29^.

A new generation of allosteric site inhibitors based on an azapeptide backbone has been described previously, where one or more amino residues are replaced by a semicarbazide group^30^. Although they are usually active site driven inhibitors^31,32^, few studies have exemplified the use of azapeptides as an allosteric inhibitors. Prostaglandin F2α (PGF2R) receptor (FP) was targeted by azabicycloalkane and azapeptide mimetics to develop a tocolytic agent for inhibiting preterm labour. The inhibitor targeted the second extracellular loop spatially distinct from the active site of FP receptor^33^. Although few studies in *P*. *falciparum* phosphoethanolamine methyltransferases (PMTs)^34^, prolyl-tRNA synthetase (ProRS)^35^ and enzymes of the non-mevalonate pathway^36^ tried to explore the role of an allosteric inhibitor in malaria, still this approach remains challenging and yet to be characterized.

Auto-processing and proteolytic maturation in proteases majorly depend on sensing the acidic environment of the specific membranous compartment. Few findings have identified and defined the role of pH sensing residues during auto-processing. Histidine residues, in general, are known to be susceptible to small changes in pH with studies reporting protonation state change from uncharged to double positively charged in acidic environment^37–39^. In Tick-borne encephalitis virus (TBEV), mutational studies have shown that pH-dependent protonation of the conserved H323 residue, located at the domain-interface DI-DIII of envelope protein was responsible for initiation of the fusion process^38^. In the human serine protease furin, H69 located in a solvent-accessible hydrophobic pocket was identified to ‘sense’ pH and initiates flexibility in the loop region of the prodomain that controls access to the catalytic site^40,41^. Likewise in histidine kinase of *Helicobacter pylori*, H94 is responsible for sensing pH and subsequent auto-phosphorylation^42^. While earlier studies have shown that falcipains auto-process in a pH-dependent manner^15,43^, an individual residue that senses pH and initiates the auto-processing event have not been identified.

Due to the pivotal role of falcipains in Hb degradation, efforts are underway to discover new modes of inhibition. Here, we present a comprehensive report describing the design and synthesis of azapeptide compounds and their effect on falcipains auto-processing. Together our biochemical and computational studies suggest that the aza-compounds are highly specific and bind to the allosteric site present in the pro-mature domain interface of falcipains. Further, we also described the role of a histidine residue in pH-dependent activation of FP3. These findings illustrate the effectiveness of targeting falcipains via an allosteric mode of action which could offer a better alternative to active site-directed inhibitors.

## Results

### Prediction of ‘hot-spot’ residues in falcipains

**Figure 1:**
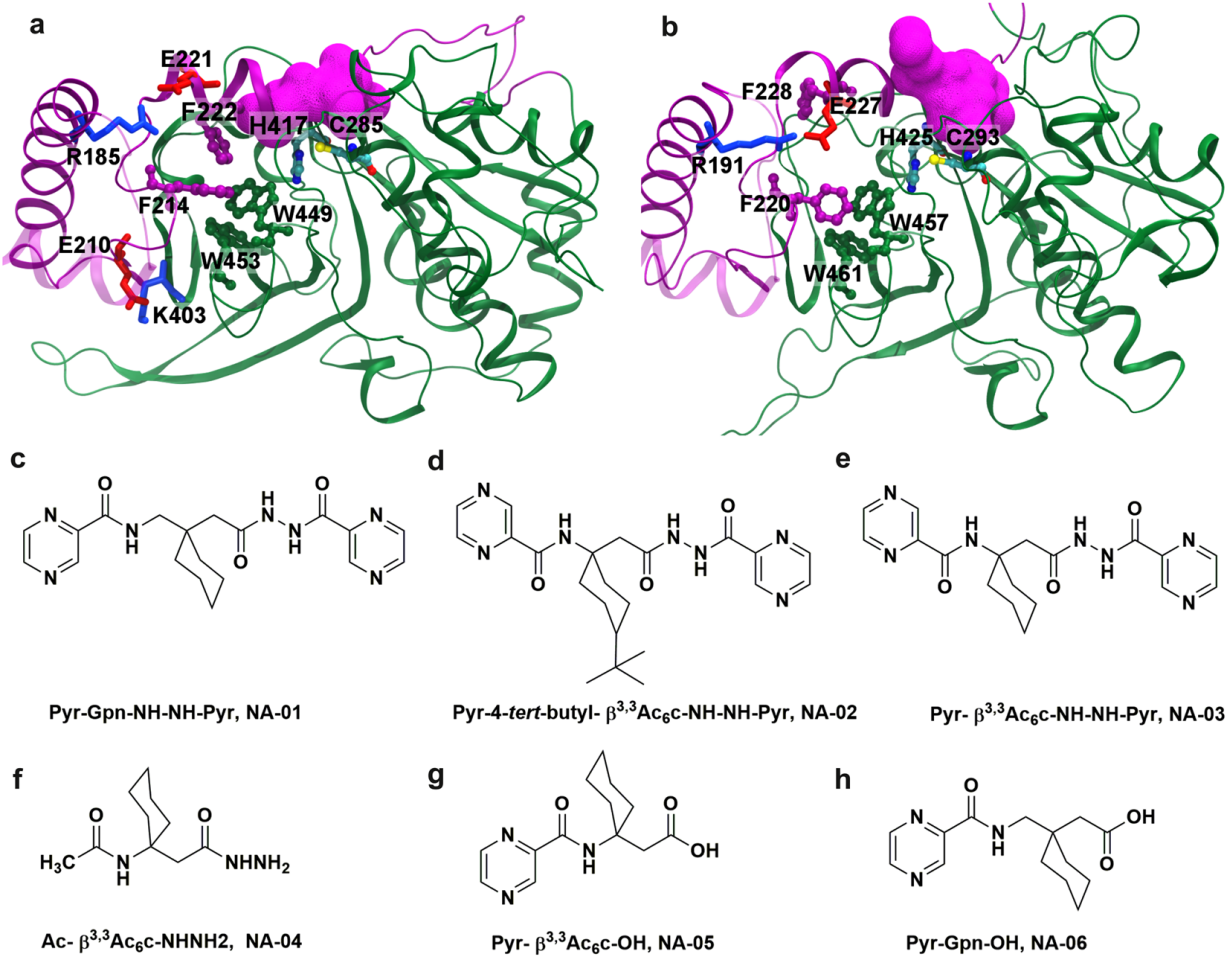
The pro-mature domain interactions in falcipains and structures of designed compounds. (**a**,**b**) The prodomain (purple) was modelled with crystal structures of the mature domain (green) in FP2 (**a**) and FP3 (**b**) Two important salt bridges R185-E221 and E210-K403 in FP2 and one salt bridge R191-E227 in FP3 were represented in pairs of red and blue. The hydrophobic cluster containing residues F214, F222, W449, and W453 in FP2; F220, F228, W45, and W461 in FP3 were illustrated in the ball-and-stick model. The catalytic site residues, C285 and H417 in FP2; C293 and H425 in FP3 were blocked by the prodomain (surface view). Synthesized compounds are as follows (**c**) Pyr-Gpn-NH-NH-Pyr, NA-01 (**d**) Pyr-4-tert-butyl-β^3,3^Ac_6_c-NH-NH-Pyr, NA-02 (**e**) Pyr-β ^3,3^Ac_6_c-NH-NH-Pyr, NA-03 (**f**) Ac-β^3,3^Ac_6_c-NHNH_2,_ NA-04 (**g**) Pyr- β^3,3^Ac_6_c-OH, NA-05 (**h**) Pyr-Gpn-OH, NA-06.

Earlier mutagenesis study has illustrated that falcipains contain crucial residues at the interface of pro- and mature domains^15^. These residues are involved in two kinds of interactions viz. salt bridges and hydrophobic interactions that are specifically involved in the auto-processing of enzymes. Based on this information, we modelled pro-enzymes and observed that residues involved in hydrophobic interactions were also the part of a hydrophobic cluster (Y207, F214, F222, Y402, F407, W449, W453) in FP2; (Y213, F220, F228, Y410, Y415, W457, W461 in FP3) and play crucial roles in enzyme activation (Fig. 1a,b). As these residues reside at the interface of the pro- and mature domains, away from the active site and influence enzyme activation, therefore, we targeted these ‘hot-spot’ residues.

### Design and synthesis of compounds

In order to target such ‘hot-spot’ residues, our candidate molecules needed to be hydrophobic in nature, so they could effectively target the hydrophobic cluster at the interface of pro- and mature domain. Additionally, these compounds have to abide by Lipinski’s rule of five to be considered as drug-like molecules^44^. Earlier studies have demonstrated that azapeptides are potent inhibitors for targeting the allosteric sites^45^. Using computational approach, we have designed and synthesized six compounds containing conformationally constrained β- and γ-amino acids with hydrophobic side chains (NA-01 to NA-06) that could target the hydrophobic pocket involving ‘hot-spot’ residues in falcipains. The chemical structures of compounds NA-01 to-NA-06 are shown in Fig. 1c–h. Both N- and C-terminus of β- and γ-amino acids are capped with a pyrazinoyl group in compounds NA-01, NA-02 and NA-03. In NA-04 and NA-05, N-terminal of β-amino acid was protected by acetyl and a pyrazinoyl moieties, respectively and C-terminus of NA-04 with a hydrazide group. In NA-06, only N-terminal of β-amino acid was protected by a pyrazinoyl group. NA-05 & NA-06, the parent compound of NA-01 & NA-03, respectively were synthesized as the negative controls. The synthetic procedure and ^1^H NMR spectra data of compounds are provided in Supplementary Section 1.1 and Supplementary Fig. S1–S6.

### Determination of the compound’s activity and viability

**Figure 2:**
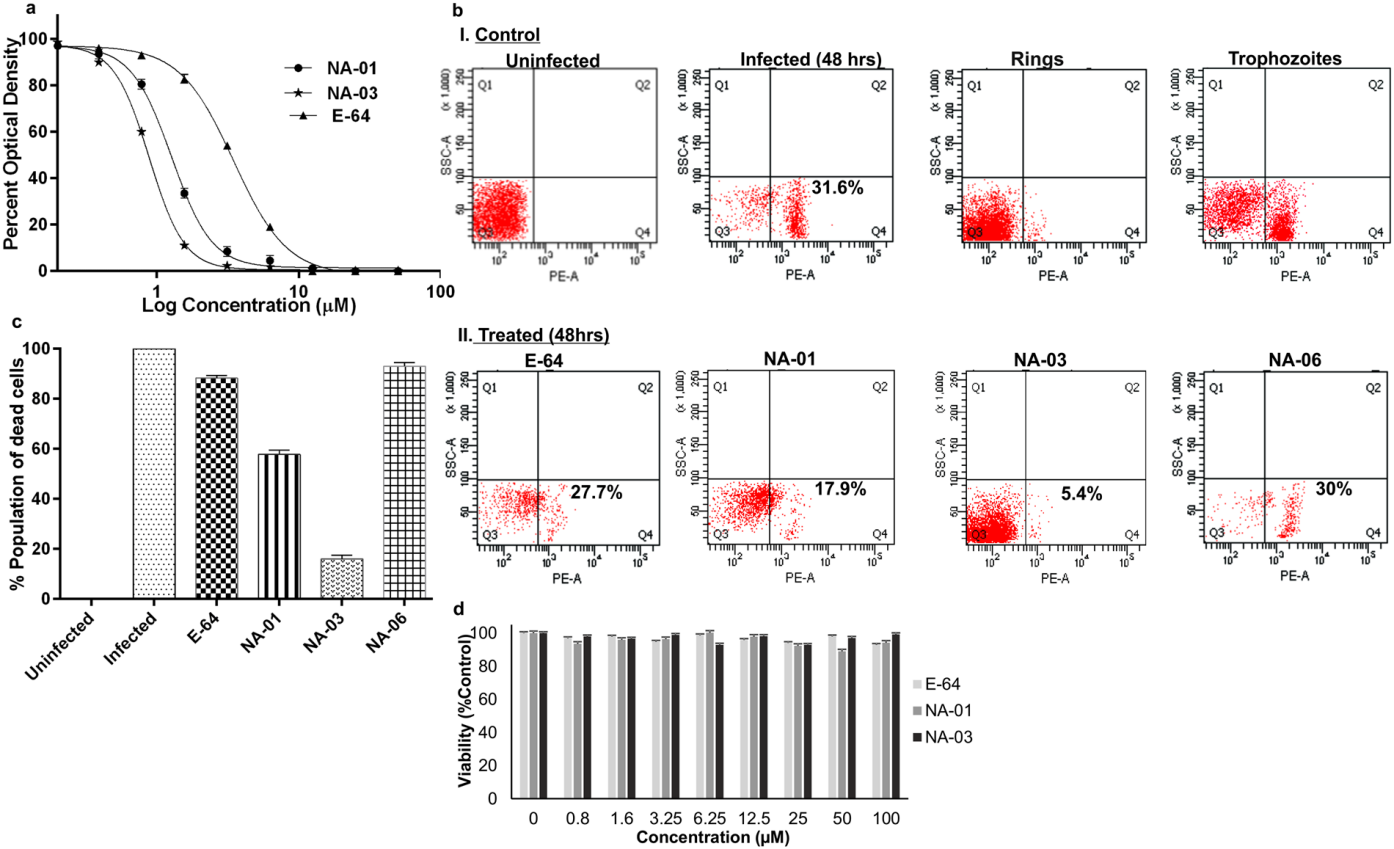
Malaria parasite growth inhibition and toxicity assay. (**a**) Dose-response of NA-01, NA-03, and E-64 verified by HRPII ELISA assay. Synchronized rings were treated with inhibitors with the concentration dilutions (1–50 μM) and the EC_50_ value was calculated by nonlinear regression analysis from triplicate measurements. (**b**) Dot plots of 48 hr untreated and compound treated parasites stained with propidium iodide and plotted as propidium iodide fluorescence relative to side scatter of white light during flow cytometry. Uninfected, 48 hr infected parasites, rings, and trophozoites were used as a control for gating purpose that allows better separation of stages to compare with inhibitor-treated parasites. The cells in the bottom right quadrant (Q4) indicated a population of the dead cells. (**c**) Bar graph representing the percentage of the dead cells population of infected RBC (iRBC) and cRBCs. The percentage of dead cells population of iRBCs was normalized to 100% (**d**) MTT assay to check the effect of E-64, NA-01, and NA-03 on PBMC after 48 hr at 570 nm. The data were presented as the mean and SD from three independent experiments.

To analyse the efficacy, compounds were incubated with synchronized ring stage of *P*. *falciparum* 3D7 parasites. Compounds NA-01, NA-02, NA-03, NA-04, NA-05, and NA-06 were dissolved in DMSO and tested for half maximal effective concentration (EC_50_) ranging from 1–50 μM in triplicates till 72 hours post infection (hpi) using HRPII ELISA assay^46,47^. The result indicated that NA-01 & NA-03 were potent inhibitors with the EC_50_ values 1.2 ± 0.6 μM and 0.8 ± 0.4 μM, respectively as compared to the EC_50_ value of E-64 was ∼4 ± 0.3 μM while DMSO did not have any effect on parasites (Fig. 2a). NA-01 & NA-03 exhibited dose-dependent inhibition whereas NA-02, NA-04, and NA-05 were screened out as they had a negligible effect. We identified that NA-03 had a five-fold better activity than E-64. For further experiments, E-64 (the active site inhibitor) and NA-06 (which lack the C-terminal pyrazine-hydrazide group) were used as positive and negative controls, respectively.

We identified the stage of parasites that were predominantly inhibited by NA-01 & NA-03. Synchronized rings and the trophozoites were stained with propidium iodide and subjected to flow-cytometric analysis^48,49^. After 48hpi, we compared uninfected RBCs (uRBCs), infected RBCs (iRBCs) and compound treated iRBCs (cRBCs) (1.2 ± 0.6 μM NA-01, 0.8 ± 0.4 μM NA-03, and 4 ± 0.3 μM E-64: based on EC_50_ values). Results showed that iRBCs had a mixed population of trophozoites and schizonts, while in case NA-01 & NA-03 treated iRBCs, growth was arrested in trophozoite stage similar to E-64. The negative control, NA-06 showed a mixed population similar to iRBCs (Fig. 2b). Further, to determine the effectiveness of compounds, we analysed the parasite clearance rate within RBC and compared the cell death rates of uRBCs, iRBCs, and cRBCs. After 48hpi, the death rate observed in control iRBCs was 31.6%, however, in presence of NA-01 & NA-03, results indicated a significant decrease in the cell death rate to 17.9% and 5.4%, respectively. E-64 showed 27.7% death rate and the negative control, NA-06 had death rates similar to iRBCs at 30% (Fig. 2b). The difference in activity of NA-03 & E-64 for parasite clearance within RBC (percentage of the dead cell) was elucidated by normalizing the value of iRBCs (31.6%) to 100% and taken as a reference for cRBCs (Fig. 2c). The combined results of the inhibitory assay and flow cytometric analysis identified NA-03 to be a potent inhibitor with five-fold better activity over E-64.

Further, we investigated the cellular toxicity of NA-01, NA-03, and E-64 using the 3-(4,5-dimethylthiazol-2-yl)−2,5-diphenyltetrazolium bromide (MTT) assay on human peripheral blood mononuclear cells (PBMC). None of the inhibitors were found to alter the PBMC levels as compared to the control group, indicated that they are selectively toxic to the parasite. All tested compounds were found to be devoid of cytotoxicity even at high concentrations (100 μM) (Fig. 2d).

### Inhibition of intra-erythrocytic development and endogenous auto-processing of falcipains

**Figure 3:**
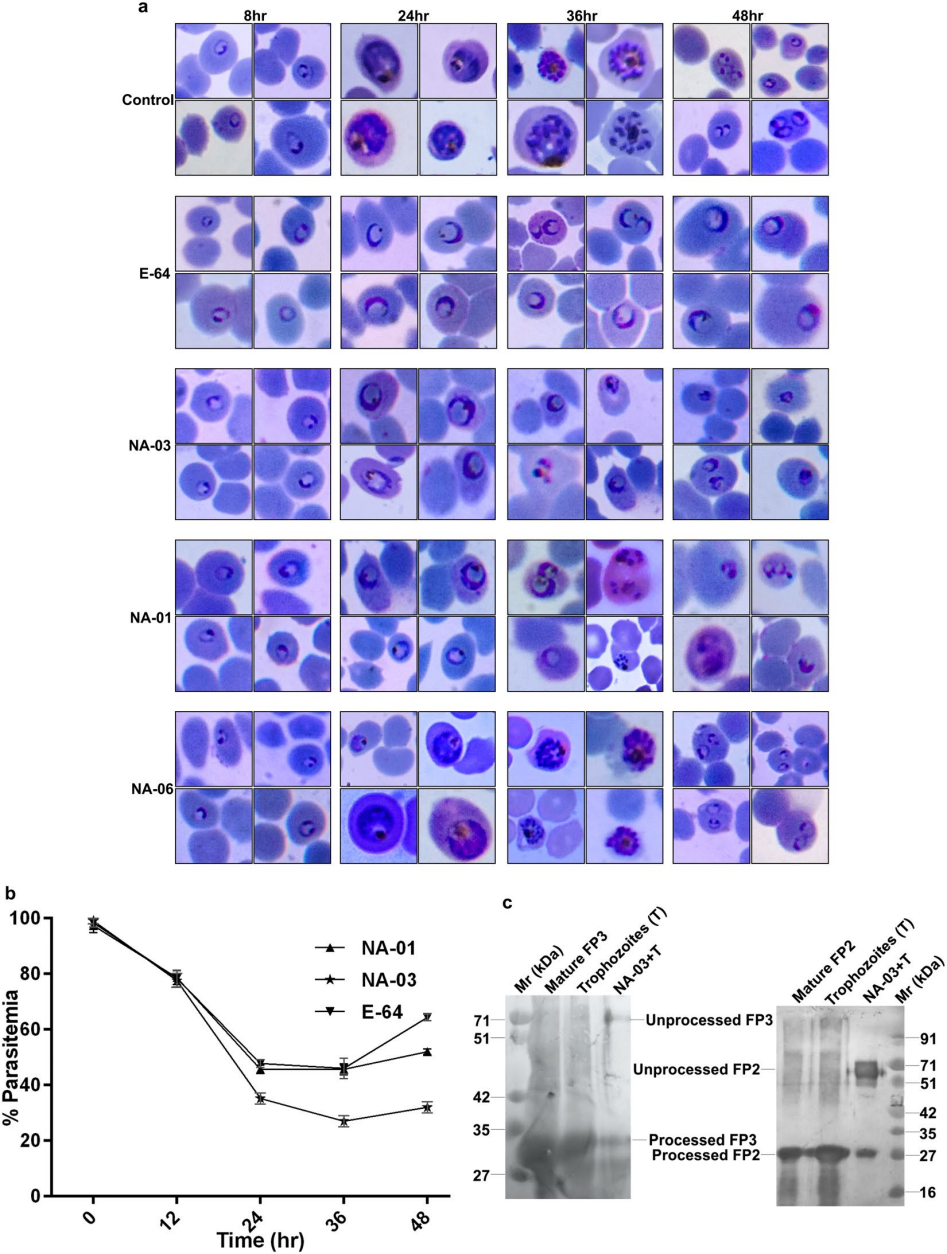
Effect of the compounds on parasite erythrocytic stages and endogenous auto-processing of falcipains. (**a**) Morphologies of inhibitor-treated parasites at 12 hr interval were observed over a period of 48 hr. Parasites were cultured in the presence and absence of 10 μM inhibitors (NA-01, NA-03, and E-64) and stained by Giemsa dye. (**b**) Percent parasitemia in the presence and absence of inhibitors were calculated for 48 hr at 12 hr intervals. Data were presented as the mean and SD from three independent experiments where control parasitemia was normalized to 100%. (**c**) Untreated and NA-03 treated trophozoites were probed using anti-FP2 and anti-FP3 antibodies, by western blot analysis. Recombinant mature FP2 and FP3 were taken as control and compared with untreated and NA-03 treated trophozoites.

To analyse the effect of inhibitors on morphology and change in parasitemia of erythrocytic stages, synchronized rings were treated with 10 μM of inhibitors (E64, NA-01, NA-03, and NA-06) at 12 hr intervals over a period of 48 hr. Control (untreated) parasites and those treated with NA-06 showed normal growth and were transited to new ring stage at the end of 48 hr (Fig. 3a). Inhibitor-treated parasites proceeded to mature ring stage at 12 hr. However, at 24 hr trophozoite stage E-64, NA-01, and NA-03 treated parasites indicated swollen food vacuole phenotype which persisted till 48 hr. In addition, NA-01 & NA-03 showed distorted schizont morphology at 36 hr followed by pyknotic effect at 48 hr. Parasite count observed during this time course indicated a significant reduction in parasitemia at 24 hr trophozoite stage (48% in E-64, 44% in NA-01 and 37% in NA-03) which remained at similar levels till 36 hr schizont stage. The transition from trophozoites to schizonts and further to rings stage were significantly reduced in NA-03, with only 30% of the total parasite population observed at end of 48 hr cycle as compared to control (Fig. 3b).

We then observed the effect of NA-03 on endogenous falcipains auto-processing, where we treated synchronized rings with NA-03, isolated 24 hr parasites and analysed for intracellular processing of pro-falcipains by immunoblotting. The untreated trophozoites showed a single band corresponding to processed falcipains, while NA-03 treated trophozoites showed two bands corresponding to unprocessed and processed falcipains, when detected with anti-falcipain antibodies. These results suggested the role of NA-03 in blocking the endogenous auto-processing of falcipains (Fig. 3c).

### Identification of the compound’s role in auto-processing

**Figure 4:**
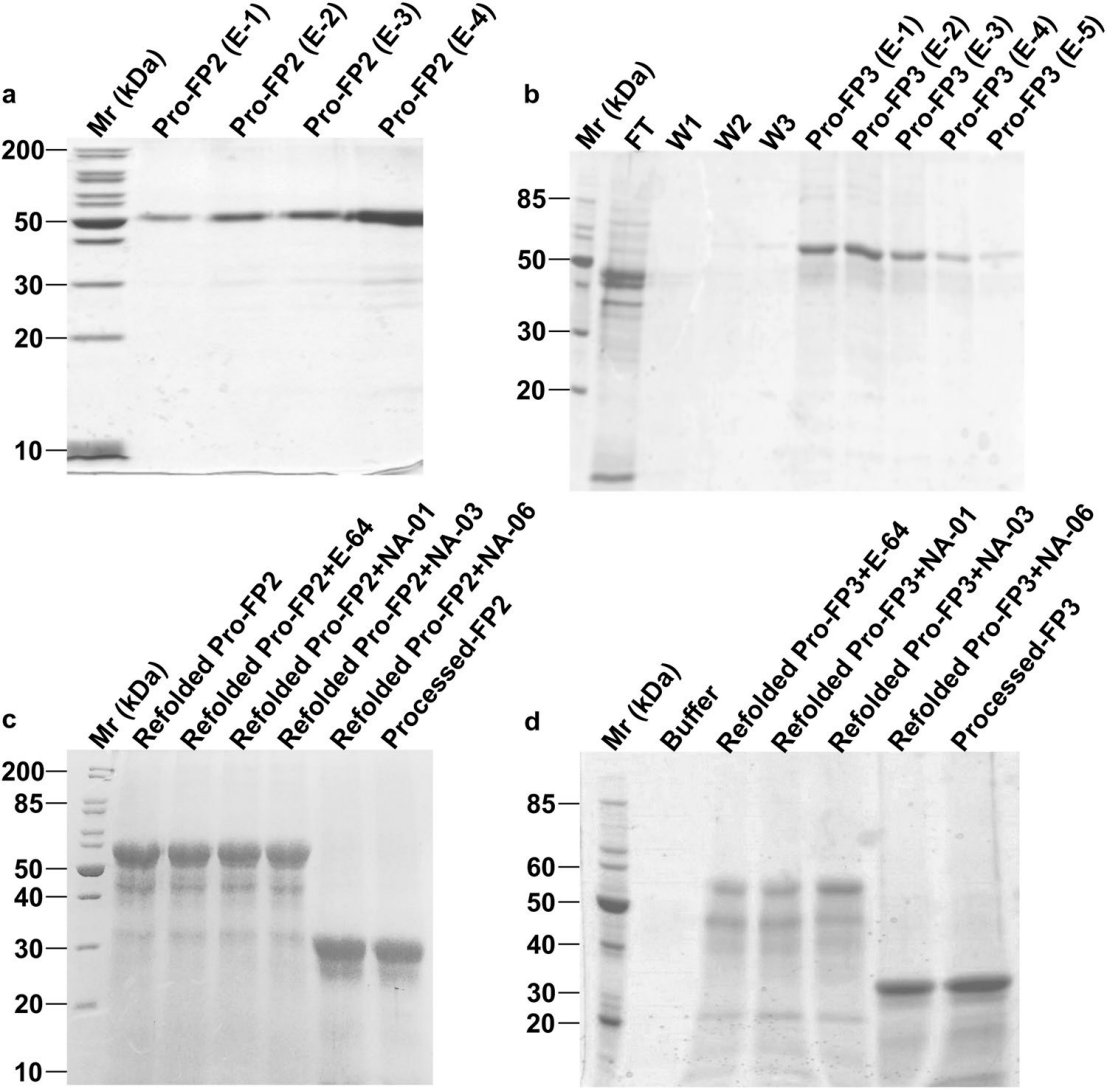
Compounds inhibit auto-processing of falcipains. (**a**,**b**) The purification profiles of pro-enzymes (whole enzymes) indicated a band sizes corresponding to 50 kDa for pro-FP2 (**a**) and 53 kDa for pro-FP3 (**b**). These enzymes were refolded (Refolded pro-enzymes) and activated under acidic conditions (pH 5.5). (**c**) Processed (Active) FP2 indicated by the cleavage of mature enzyme showing a band of 27 kDa (lane 7) while auto-processing of FP2 was blocked in the presence of inhibitors NA-01, NA-03, and E-64 indicated by the band of 50 kDa. (**d**) Similarly, for pro-FP3, the activated enzyme was indicated by a band of 27 kDa (lane 7) while in the presence of inhibitors NA-01, NA-03, and E-64, the auto-processing was blocked indicated by a band of 53 kDa. A negative control, NA-06 did not have any effect on enzymes processing (**c** lane 6; **d** lane 6). All results were analysed using 12% SDS-PAGE.

To determine the effect of compounds during auto-processing, pro-enzymes (whole enzyme): pro-FP2 (50 kDa) and pro-FP3 (53 kDa) were expressed, purified and refolded as described earlier^6,10^ (Fig. 4a,b). When refolded pro-enzymes were activated under acidic conditions, get directly processed to 27 kDa active enzymes (Processed FP2, FP3) (Fig. 4c lane 7; 4d lane 7). However, refolded pro-enzymes after incubated with NA-01, NA-03, and E-64 for 10 min, and activated under acidic conditions, pro-enzymes were unable to auto-process and remained intact (Fig. 4c,d). NA-06 did not have any effect on pro-enzymes as they processed directly to active enzymes (Fig. 4c lane 6; 4d lane 6).

### Determination of the compound’s binding site

**Figure 5:**
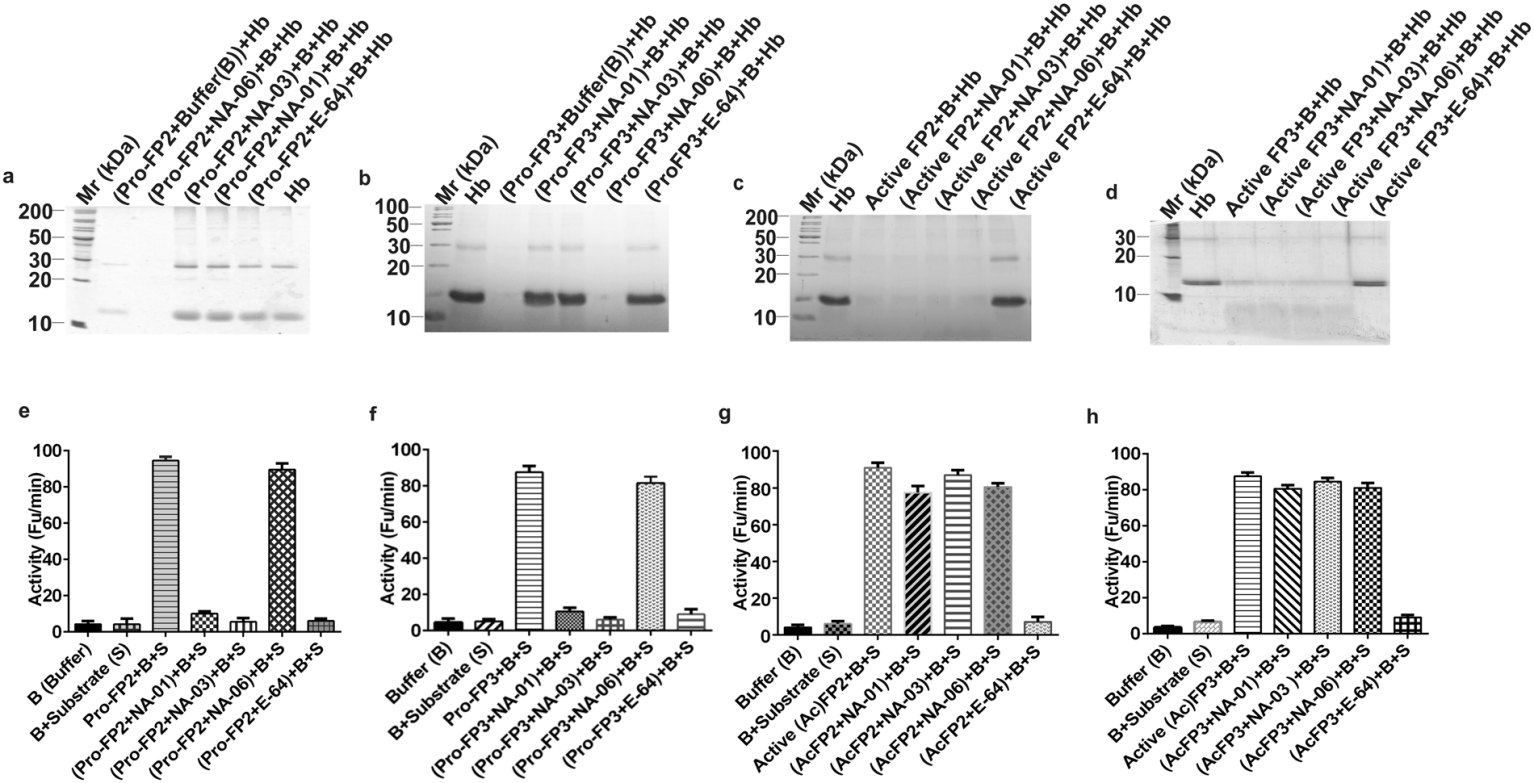
Compounds do not bind active site. (**a**,**b**) Refolded pro-enzymes pro-FP2 (**a**) and pro-FP3 (**b**) incubated with inhibitors and activated under acidic condition. Pro-enzymes were unable to hydrolyze Hb in the presence of NA-01, NA-03, and E-64, unlike negative control, NA-06. (**c**,**d**) However, pro-enzymes when refolded and activated under acidic conditions released processed enzyme: active FP2 (**c**) and active FP3 (**d**) that were able to hydrolyze Hb effectively even in the presence of inhibitors. (**e**,**f**) Refolded pro-enzymes pro-FP2 (**e**) and pro-FP3 (**f**) when incubated with inhibitors and activated under acidic conditions, were unable to cleave fluorogenic substrate Z-L-R-AMC, unlike NA-06 (negative control). (**g**,**h**) However, pro-enzymes when refolded and activated, generated active FP2 (**g**) and active FP3 (**h**) that cleaved the fluorogenic substrate even in the presence of inhibitors, unlike E-64.

To identify the binding site, we incubated refolded pro-enzymes with inhibitors NA-01, NA-03, NA-06, and E-64 for 10 min followed by activation under acidic conditions. Their enzymatic activities were tested by Hb hydrolysis and fluorogenic substrate assays. The results showed that in presence of the inhibitors, NA-01, NA-03, and E-64, the pro-enzymes remained intact and thus could not cleave the substrates (Fig. 5a,b,e,f), while in the presence of NA-06, pro-enzymes underwent auto-processing and released active enzymes that in turn hydrolysed the substrates (Fig. 5a lane 3; 5b lane 6; 5e lane 6; 5f lane 6). These data confirmed that the presence of prodomain was required for effective binding of inhibitors NA-01 & NA-03.

To eliminate the possibility of being active site inhibitors, we treated activated (processed) FP2 and FP3 with inhibitors NA-01, NA-03, NA-06, and E-64 and determined enzymatic activity using Hb and fluorogenic substrate assays. Results of substrate assays revealed that unlike E-64, inhibitors NA-01, NA-03, and NA-06 did not affect falcipain’s activity (Fig. 5c,d,g,h) indicating that compounds NA-01 & NA-03 were not the catalytic site inhibitors.

### Structural analysis and biomolecular interaction of an inhibitor with pro-enzyme

**Figure 6:**
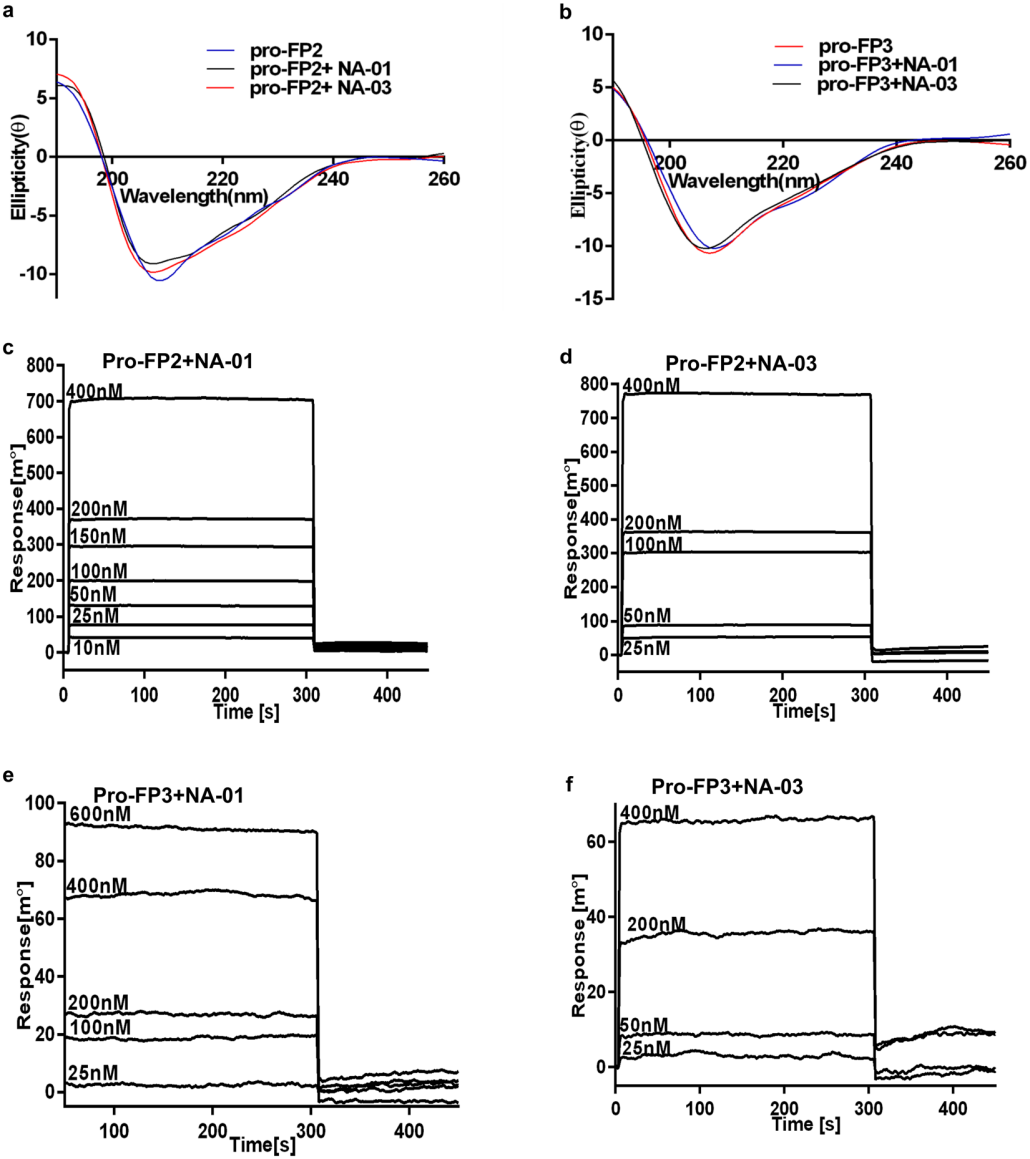
Circular dichroism and kinetic analysis of pro-enzymes in the presence of inhibitors. (**a**) Pro-FP2 alone (blue), pro-FP2 + NA-01 (black) and pro-FP2 + NA-03 (red) were incubated in 10 mM phosphate buffer, pH 8.0. (**b**) Pro-FP3 alone (pink), pro-FP3 + NA-01 (blue), pro-FP3 + NA-03 (black) were incubated in 10 mM phosphate buffer, pH 8.0. The absorbance of CD spectra were measured between 190–260 nm after an average of five best scans. (**c**,**d**) pro-FP2 coupled to flow cell was equilibrated and increasing concentrations of NA-01 (**c**) and NA-03 (**d**) were injected over the sensor chip. (**e**,**f**) Pro-FP3 coupled to flow cell was equilibrated and increasing concentrations of NA-01 (**e**) and NA-03 (**f**) were injected over the sensor chip.

Circular Dichroism (CD) spectroscopy was used to probe the secondary structure of pro-enzymes in the presence and absence of inhibitors, NA-01 & NA-03. CD spectra clearly demonstrated that the inhibitors did not alter the secondary structures of pro-enzymes. Pro-FP3 showed 21% α-helices, 28% β-sheets and 52% random coils as compared to pro-FP3 in presence of NA-01 which exhibited 23% α-helices, 27% β-sheets and 52% random coils, while pro-FP3 with NA-03 displayed 21% α-helices, 26% β-sheets and 53% random coils (Fig. 6b) using Dichroweb software. Similar results were obtained with pro-FP2 alone and in the presence of NA-01 & NA-03 (Fig. 6a). The pro-enzyme-inhibitor interactions were verified by surface plasmon resonance (SPR) based on biomolecular interaction analysis. Purified pro-enzymes (pro-FP2 and pro-FP3) were immobilized to an SPR gold chip by amine coupling chemistry^11^. Different concentrations of inhibitor were injected into a chip immobilized with pro-enzymes, and the binding affinity was measured in resonance units (RU). Results suggested that inhibitors, NA-01 & NA-03 had a dose-dependent increase in SPR response. The binding constant (K_d_) of NA-01 & NA-03 for pro-FP2 were 1.72 ± 0.03 nM, 1.67 ± 0.01 nM, respectively (Fig. 6c,d). Similarly, the K_d_ values of NA-01 & NA-03 for pro-FP3 were 5.14 ± 0.07 nM and 2.0 ± 0.04 nM, respectively (Fig. 6e,f).

### NA-03 is an allosteric inhibitor of FP2

NA-03 could not inhibit haemoglobin hydrolysis after falcipains activation, therefore it was expected to bind either at the interface of the pro-and mature domain (residue 155 to 244) or within the prodomain to prevent activation associated structural reorganization. To determine NA-03 binding site in FP2, we first modelled a prodomain structure in the presence of a mature domain, and subsequently performed 5 × ∼130 ns molecular dynamics (MD) simulations to monitor the conformational equilibration (see Supplementary Sections 2.1–2.2, and Supplementary Fig. S4). The simulation results suggest that the modelled segment had a negligible impact on the mature domain conformations, hence, we considered the equilibrations to be sufficient for the further determination of binding site (see Supplementary Section 2.2).

**Figure 7:**
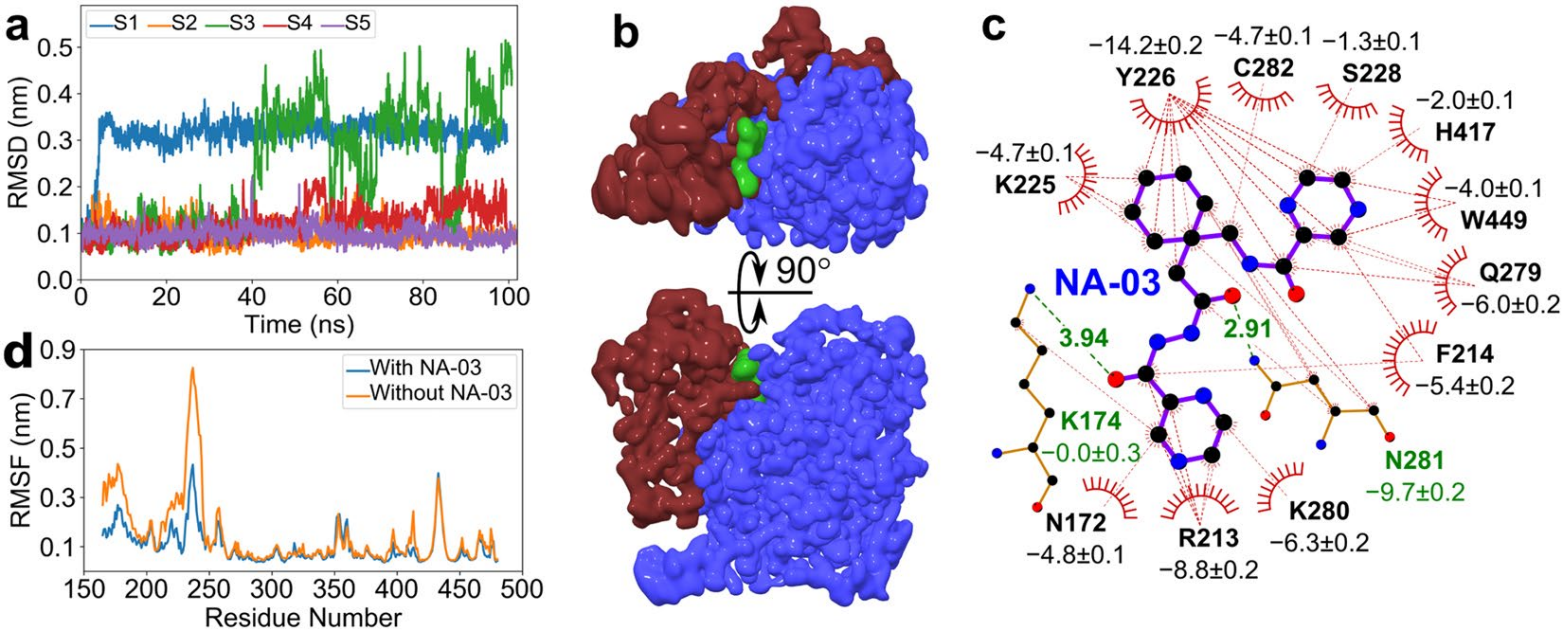
NA-03 dynamics and impact of NA-03/FP2 interaction on pro-FP2 flexibility. (**a**) RMSD of NA-03 with a reference to starting conformation as a function of time during five equilibrium. MD simulations (denoted by S1, S2, S3, S4, and S5). The RMSD values were calculated after superposition of the mature domain on the starting structure. (**b**) Average 3D atomic density of pro- (red) and mature (blue) domains with bound NA-03 (green) at the interface. The densities were calculated from five combined MD trajectories. (**c**) Interactions network of NA-03 with FP2. Interaction energies (kJ/mol) contributing to binding calculated with the MM-PBSA method from combined trajectories using g_mmpbsa^59^. Red dashed lines indicated van der Waals and hydrophobic interactions while green dashed lines showed potential hydrogen bond interactions. (**d**) Impact of NA-03 binding on FP2 flexibility. RMSFs of FP2 residues are shown as a function of residue number in the presence (blue) and absence (orange) of NA-03.

Next, pro-FP2 conformations were used to determine the NA-03 binding site. For this aim, we designed a novel computational protocol comprising ensemble molecular docking, MM-PBSA rescoring and accelerated ligand sampling MD simulations (see Supplementary Fig. S5). The protocol was employed on NA-03 with FP2, and the final NA-03/FP2 complex was selected by considering both binding energies and FP2 conformational fluctuations (see Supplementary Section 2.3). Further, the final NA-03/FP2 complex was validated by monitoring equilibration of NA-03 conformations during 5 × 100 ns equilibrium MD simulations (see Supplementary Section 2.4). As shown in Fig. 7a, root mean square deviation (RMSD) of NA-03 with respect to its starting structure remained quite low during ∼75% of the simulation time. This result was further corroborated by the average 3D atomic density maps calculated from combined MD trajectories (Fig. 7b). During the remaining ∼25% of the time, NA-03 remained within the cavity with slight conformational changes. Overall, NA-03 tends to remain inside the binding cavity throughout the simulations with conformational fluctuations (see Supplementary Fig. S8). Therefore, we considered this modelled complex comparable to the physiological native conformation and performed further studies.

Further, we examined important residues that were favourable for NA-03 binding. An interaction network was generated for all residues that were within 4 Å of NA-03 (Fig. 7c, see Supplementary Section 2.4.1). The result showed that NA-03 interacted with hydrophobic, W449, polar N172, Q279, N281, C282, charged R213, K225, and K280 residues of FP2. Particularly, prodomain R213 guanidinium group formed a pi-cation interaction with NA-03 pyrazine ring. Additionally, prodomain’s N172 interacted with the pyrazine ring in NA-03. Interestingly, mature domain K280 residue also interacted with the pyrazine ring (Fig. 7c). Moreover, two hydrophobic residues (F214 and Y226) in the prodomain interacted with the middle phenyl group, which also interacted with the mature domain’s N281 and C282 residues. Furthermore, both pro- and mature domains interacted with NA-03 pyrazine rings through F214, S228, Q279, H417, and W449 residues.

Remarkably, we observed a significant reduction in the binding of NA-05 and NA-06 inhibitors due to the absence of hydrazide-pyrazine group that abolished interaction with N172, R213, K280, and N281 residues in FP2 (Fig. 7c, see Supplementary Fig. S9). This observation strongly validated our simulation result and the obtained interaction network. Additionally, earlier studies indicated that mutations of F214, W449, and W453 residues inhibited FP2 activation^15^, therefore, corroborating our computationally determined NA-03 binding site. Overall, the combined simulation results and experimental observations showed that NA-03 most likely binds at the interface and interact with both the pro- and mature domain.

### NA-03 resists prodomain structural reorganization

We determined whether FP2 and NA-03 interaction network inhibited the enzyme activation process and whether the network could resist prodomain conformational reorganization associated with the FP2 activation process. To test this assumption, we calculated residue’s root mean square fluctuations (RMSF) with a reference to a starting structure both in the presence and absence of NA-03 during equilibrium MD simulations and analysed the induced rigidity in FP2, after the NA-03 binding.

We observed reduced RMSF values of the prodomain upon NA-03 binding, particularly in the regions spanning 165 to 185 and 210 to 240 residues (Fig. 7d). These regions contain residues N172, R213, F214, K225, and Y226 that are also part of the interaction network (Fig. 7c). This result suggested that the NA-03 interaction impedes the structural fluctuations and induces rigidity in FP2 prodomain. Moreover, these interacting residues from the prodomain did not contain any negative charged residue or histidine, and therefore, an impact of pH reduction on these interactions was likely to be negligible due to constant protonation state.

### Histidine-dependent activation of FP3

**Figure 8:**
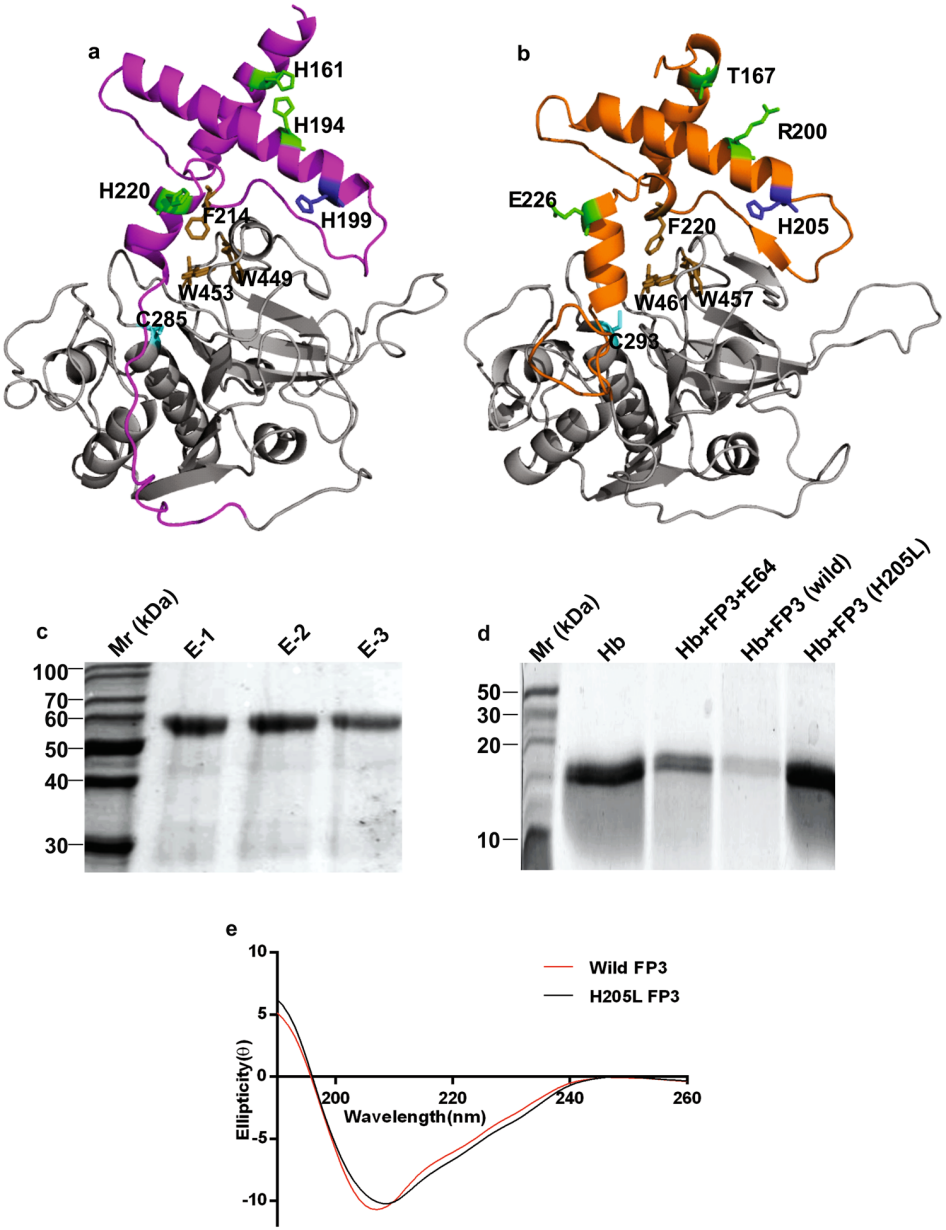
Role of a histidine residue in FP3 activation. (**a**) FP2 and (**b**) FP3 prodomain have four histidine residues, among them H205 (blue) in FP2 prodomain (purple) and H199 (blue) in FP3 prodomain (orange) is highly conserved. Rest of three histidine residues positions H161, H194, and H220 in FP2 and T167, R200, and E226 in FP3 are differentially conserved represented in green sticks. The mature domain containing a catalytic cysteine (cyan) C285 in FP2 and C293 in FP3 and hydrophobic interaction residues (brown) F214, W449, W453 in FP2 and F220, W457, W461 in FP3 are also highlighted. (**c**) The purification of H205L-FP3 mutant indicated a band of 53 kDa. (**d**) Hb hydrolysis assay showed that wild FP3 effectively hydrolyze the substrate, unlike H205L-FP3 mutant and an inhibitor, E-64. (**e**) CD spectra exhibiting wild FP3 (red), H205L-FP3 mutant (black) incubated in 10 mM phosphate buffer, pH 8.0. The absorbance of spectra were measured between 190–260 nm after an average of five best scans.

The role of histidine residues in pH-dependent activation of proteases have been investigated in other organisms, although this approach remains undetermined in malarial cysteine proteases. Multiple Sequence Alignment (MSA) analysis of cysteine proteases from different organisms revealed that prodomain contains four conserved histidine residues (see Supplementary Fig. S10). While three of these residues are differentially conserved among FP2 and FP3, the fourth highly conserved residue H199 in FP2 and H205 in FP3 lies near the loop containing the conserved GNFD motif (Fig. 8a,b). GNFD motif has an important role in pro-mature domain interactions, thereby in enzyme activation^15^. Studies in furin protease showed that substitution of conserved histidine at 69 positions to leucine (H69L) completely blocked the ability of acidic pH to trigger furin activation. Thus, we explored the role of this conserved histidine through substitution with a residue of opposing properties. Histidine is a polar residue, therefore a non-polar residue of similar size was selected for substitution. We designed a mutation at 205 position and replaced the conserved histidine to leucine (H205L), and studied its effect on FP3 activation.

Mutant H205L was expressed and activated similar to wild pro-FP3 and a band of 53 kDa was observed^6,10^ (Fig. 8c). CD analysis of wild and mutant showed no significant changes in secondary structure, wild pro-FP3 exhibited 21% α-helices, 28% β-sheets and 52% random coils, mutant H205L indicated 15% α-helices, 30% β-sheets and 55% random coils (Fig. 8e). However, Hb hydrolysis assay indicated that wild FP3 efficiently degraded Hb, while H205L mutant did not hydrolyse Hb, suggesting that mutation blocked the histidine-dependent activation of FP3 (Fig. 8d).

## Discussion

Within the Hb degradation pathway, falcipains are the key regulators that facilitate parasite growth and metabolism. Falcipains are synthesized as zymogens and have unique pro- and mature domain linked via a salt bridge and hydrophobic interactions^15^. When falcipains encounter a compartment-specific pH changes, initial breakdown of hot-spot interactions occur followed by detachment of the prodomain, leading to auto-processing and activation of the enzyme. In this study, we demonstrated a novel mechanism of an allosteric site inhibition at the interface of pro- and mature domain that blocked the auto-processing of falcipains. We designed and synthesized six azapeptide based compounds containing conformationally constrained β- and γ-amino acids and monitored their effect on parasite asexual stages. Two potent compounds NA-01 & NA-03 showed dose-dependent inhibition *in vitro*, both were highly specific to falcipains and did not show off-target activity against human PBMCs. NA-03 exhibited five-fold better activity compared to an active site inhibitor, E-64 of falcipains.

During intra-erythrocytic development of parasites, a significant reduction in parasitemia at trophozoite stage was observed. This was likely due to the accumulation of undegraded Hb in the food vacuole^6^, indicated inhibition of falcipain by NA-03. The transition from trophozoite to schizont and further ring stages in NA-03 treated parasites was manifested by distorted parasite morphology which persisted till 48 hr, signifying growth arrest. Thus, NA-03 blocked the development of erythrocytic stages, predominantly trophozoites, and schizonts, caused by inhibition of FP2 and FP3. Auto-processing of endogenously synthesized pro-FP2 and pro-FP3 was inhibited by NA-03, however, only partial inhibition was observed as the falcipains processing mainly occurred in a cellular compartment, which was not readily accessible to the inhibitor. A previous study also suggested that the processing occurs in a cellular compartment after transport via ER, and that compartment was relatively inaccessible to the inhibitors which are less lipophilic in nature^50^. Therefore, the lipophilicity of the compounds may be increased in near future for higher potency.

The auto-proteolytic activity of recombinant pro-enzymes was completely blocked in presence of NA-01 & NA-03, similar to E-64. The inhibitors, once bound at the interface of the pro-mature domain, blocked the ‘hot-spot’ interactions and did not allow dissociation of prodomain, thereby restricting the auto-processing. In turn, no active enzymes were available to hydrolyse either their natural or fluorogenic substrates. However, activated enzymes even in the presence of NA-01 & NA-03, were capable of hydrolysing the substrates, and indicated that the inhibitors did not bind to the catalytic site. These inhibitors showed a dose-dependent affinity for the proenzymes without any change in the secondary structure. Together, these results confirmed that compounds NA-01 & NA-03 did not inhibit the active site but rather blocked the auto-processing event when falcipains exist in their pro-forms.

Determination of NA-03 binding site in FP2 at a molecular resolution exhibited some interesting insights. Firstly, NA-03 remained inside FP2 and retained the similar conformation ∼75% of the simulation time, similar to the initial state. Secondly, we identified important residues in FP2 that interact with NA-03 from the simulation trajectories. The obtained interaction network suggested that NA-03 bound to FP2 in a particular orientation, where its entire structure interacts with both pro- and mature domain residues to prevent auto-processing. The negative control, NA-06 showed a significant reduction in its binding affinity due to the absence of pyrazine-hydrazide group as compared to NA-03. Finally, this observation validated our simulation results and explained the reduced binding affinity of NA-06. Additionally, simulation results showed a marked decrease in prodomain flexibility upon NA-03 binding, suggesting that it resists the structural reorganization irrespective of the pH reduction, and thus blocked auto-processing and further enzyme activation. Furthermore, docking experiments revealed that E-64 bound near the catalytic site (C285 and H417) of activated FP2 and its spatial position coincided with structurally similar S228-L229-R230 residues of prodomain in FP2 (see Supplementary Fig. S10). Thus, it is highly unlikely that any ligand would occupy the active site cavity in presence of the prodomain because it has to displace S228-L229-R230 residues from the cavity. In contrast, NA-03 is expected to bind at the interface of pro- and mature domain to prevent structural reorganization as obtained from the simulation results (Fig. 7). Therefore, NA-03 most likely acts as an allosteric inhibitor (see Supplementary Fig. S10).

Further, we demonstrated histidine-dependent activation in FP3 by a mutagenesis approach. As FP2 and FP3 are biochemically similar with 63% identity and FP3 proved to be essential for the parasite survival, therefore, in our study we selected FP3 for mutagenesis. Earlier studies in human protease, furin, showed the role of a conserved histidine residue in pH sensing based activation of the enzyme. Therefore, we selected a conserved, polar residue, histidine at 205 position and substituted it with leucine (H205L), a nonpolar residue with similar size to histidine. FP3 H205L mutant was unable to generate functional active enzyme even under acidic activation conditions. Although whole FP3 and H205L mutant had similar secondary structures, H205L mutant could not degrade Hb. Our preliminary bioinformatics and experimental data support the notion of the histidine based pH ‘sensing’ mechanism of falcipains activation. However, the detailed mechanism of histidine regulated activation in cysteine proteases may further be deduced by performing crystallography and MD simulations.

Though there has been evidence indicating a redundancy in the Hb degrading pathway^51,52^, falcipains could still be considered as attractive drug targets. While disruption of FP2 resulted in parasite growth arrest at trophozoite stage, disruption of FP3 could not even be achieved, indicating their importance as haemoglobinases^6,7^. Till date, the general strategy for targeting proteases involved active site inhibition, which led to the development of specific but less selective inhibitors. However, the active site being highly conserved would be confronted by high drug pressure, thus would be more susceptible to mutations which resist inhibitor interactions, as exemplified in HIV^53,54^ and malaria^55,56^. Few studies have identified and characterized inhibitors that target allosteric sites, which are relatively more selective, potent and have fewer side effects^57^. Allosteric sites could offer higher selectivity due to the formation of a flexible binding cavity, allowing the ligand to adopt multiple conformations for co-operative inhibition. When an inhibitor binds at the allosteric pocket, it maintains the enzyme in a low energy conformation (preferentially zymogen state) and results in little to no activity. Our study demonstrated that inhibitor binds at the ‘hot-spot’ interactions and maintains the naturally favoured zymogen state of falcipains. This is the first report to elucidate the blocking of an auto-processing event in falcipains via an allosteric mechanism. Targeting allosteric sites in falcipains can serve as a new mechanism-based approach in malaria, which could be less prone to drug resistance.

## Methods

### Ethical statement

PBMCs were isolated from human blood of the healthy individuals after signing an informed consent form approved by the institute (ECR/NIMR/EC/2015/251). The parasite culture work was conducted at Malaria Parasite Bank of ICMR-National Institute of Malaria Research (NIMR), New Delhi, has been approved by the ethical committee for *in vitro* culture of *P*. *falciparum* (ECR/65/Inst/DL/2013). All the methods were carried out in accordance with the guidelines and regulations approved by the Institutional Ethical Committee of NIMR.

### *In-vitro* parasite cultivation and drug sensitivity assay

*P*. *falciparum* 3D7 culture was maintained in human erythrocytes (procured from the Indian Red Cross Society) at 2% haematocrit in RPMI 1640 (Gibco, Life Technologies) supplemented with 41.1 mg/litre hypoxanthine, 300 mg/l glutamine, 5% sodium bicarbonate, 10% human serum and maintained in 90% N_2_, 5% CO_2_, and 5% O_2_. Ring stage synchronization was maintained by 5% D-sorbitol. 96 well ELISA plates were dosed in triplicates with 50 μM of each inhibitor (NA-01, NA-02, NA-03, NA-04, NA-05, and NA-06) dissolved in 1% DMSO and was serially diluted six-fold. A cell medium mixture (CMM) was prepared by adding 0.94 ml of the parasitized blood (1%) to 24.06 ml of RPMI-1640 medium in a sterile disposable tube. 200 μl of CMM was added to each well and incubated for 72 hr at 37 °C in CO_2_ incubator (Flow Laboratories).

For HRPII ELISA Assay, 96 well ELISA plates were initially pre-coated with 100 μl of 0.1 μg/mL primary IgM antibody MPFM-55A (Immunology Consultants Laboratories, Inc.) and incubated at 4 °C overnight. Plates were dried and blocked with 200 μl of 2% bovine serum albumin (BSA) in blocking solution, PBS (Phosphate Buffer Saline), and washed thrice with PBS-Tween 20 (0.05%). Plates were stored at −20 °C till further experiment was conducted. After 72 hr 100 μl of a haemolysed lysate of parasite culture was transferred to the pre-dosed primary IgM antibody-coated plate and incubated at room temperature (RT) for 1 hr. The content was discarded and plates were washed thrice with PBS-Tween 20 (0.05%), followed by addition of 100 μl of 0.2 μg/mL secondary IgG antibody MPFG-55P (Immunology Consultants Laboratories) dissolved in 2% BSA and 1% Tween 20. IgG added plate was incubated for 1 hr at RT, washed thrice in PBS/Tween and dried. 100 μl 3,3′,5,5′-Tetramethylbenzidine (TMB) chromogen (Amresco) was added to each well and incubated in dark for 10 min at RT. The reaction was stopped by addition of 50 μl of 1 M sulphuric acid and absorbance of each plate were recorded using an ELISA plate reader (Spectrostar Nano, BMG Labtech) at 450 nm^46,47^. The EC_50_ was calculated by nonlinear regression analysis from triplicate measurements. The software used for the study is based on a polynomial regression model and is freely available from http://malaria.farch.net and graphs were prepared using GraphPad Prism.

### Stage-specific inhibition of *P*. *falciparum* 3D7 strain using flow cytometry

Synchronized ring stage was treated with the inhibitors (1.2 μM NA-01, 0.8 μM NA-03, 4 μM E-64; based on obtained EC_50_ values) in triplicates and incubated at 37 °C in CO_2_ incubator (Flow Laboratories). After 48 hr, the plate was centrifuged at 3000 rpm for 15 min, the supernatant was discarded and 100 μl (0.05% glutaraldehyde (Sigma) in 1X PBS) was added to the pellet for 20 min at RT. The fixed cells were permeabilized with 0.25% Triton X-100 in PBS for 5 min at RT and stained with a 1:100 dilution of a 5 mg/ml working solution of propidium iodide (Sigma) in deionized water as described earlier^48,49^. Synchronized ring and trophozoite stages were taken as a reference and compared to the compound treated RBC (cRBC) after 48 hpi. Percent populations of the dead cells were calculated by normalizing iRBC populations to 100% and used as a reference to calculate the cRBC population. The parasite life cycle progression was monitored by using a BD FACScan flow cytometer (Becton, Dickinson, and Co.).

### Measurement of Cytotoxicity for human Peripheral Blood Mononuclear Cells (PBMCs)

10 ml of human blood was collected and mixed with the same amount of complete medium (RPMI 1640 with 10% human serum) and layered over an equal volume of Histopaque-1077 (Sigma). After 20 min, the mixture was centrifuged at 400 g at RT, the interphase containing PBMCs was collected and transferred to a fresh tube. Again, mixed with 10 ml complete medium and centrifuged at 350 g for 10 min. The cells were washed twice with 10 ml complete medium. The washed cells were suspended in complete medium (5.5 × 10^5^ cells/ml) and incubated at 37 °C in a CO_2_ incubator. After 20 hr, the cells were suspended in fresh 100 μl culture medium, transferred to 96 well plate and incubated at 37 °C in a CO_2_ incubator. After 4 hr, inhibitors (NA-01, NA-03, and E-64) with concentration ranging from 1–100 μM in triplicates were added to the plate and incubated at 37 °C in a CO_2_ incubator for 48 hr. Cytotoxicity was determined by MTT (Sigma). Briefly, 20 μl MTT (5 mg/ml) in HBSS (Hanks’ balanced salt solution, cell clone) was added and the plate was incubated at 37 °C in a CO_2_ incubator for 3 hr. The plate was centrifuged at 894 g for 5 min, the supernatant was removed and the cells were washed with 150 μl of HBSS. The plate was centrifuged again at 894 g for 5 min, and the supernatant was removed. The insoluble MTT product formazan was solubilized with 150 μl DMSO in each well, and absorbance was measured at 570 nm with spectrophotometer Nano Quant infinite M-200PRO (Tecan). The data were presented as the mean and SD from three independent experimental values.

### *In vitro* malaria parasite growth inhibition assay

Highly synchronized ring stage parasites (3% rings) were grown for 48 hr in presence of 10 μM of inhibitors (E64, NA-01, NA-03, and NA-06). Blood smear for parasites were prepared after every 12 hr, stained with Giemsa (HiMedia) and observed under a 100x objective lens compound microscope. In parallel, the values of untreated and inhibitor-treated parasites were compared to determine the parasite growth and level of parasitemia. At least 20 cells per field were observed to assess the parasite morphology and similar fields were counted to determine the parasitemia.

### Effect of the inhibitor on the endogenous processing

Synchronized rings with 10% parasitemia were treated with NA-03, after 24 hr at trophozoite stage, the parasites were collected and isolated. The culture was centrifuged at 894 g for 5 min, the supernatant was discarded, and the pellet was treated with ice-cold 0.01% saponin (in PBS) for 5 min to lyse the erythrocytes. Further the pellet was centrifuged at 12,096 g for 5 min at 4 °C. Again the supernatant was discarded, the pellet was washed twice with ice-cold PBS to remove residual Hb, and parasites were recovered by centrifugation at 12096 g for 5 min at 4 °C. Isolated parasites were dissolved in PBS containing a cysteine protease free cocktail inhibitor (Sigma Aldrich) and sonicated at 90 sec pulse rate (BR Biochem Life Sciences) and subjected to three freeze/thaw cycles and centrifuged at 12096 g for 15 min at RT. The supernatant was collected and suspended in 5x reducing SDS-PAGE sample buffer, heated at 90 °C for 10 min and centrifuged at 12096 g for 15 min at RT. Untreated and NA-03 treated trophozoites along with recombinant FP2 and FP3 were analysed on 10% SDS-PAGE, and the resolved proteins were transferred onto an Immobilon-P PVDF Membrane (Millipore). The membrane was blocked with 5% skimmed milk in 1X PBS for 2 hr and washed twice with PBS for 10 min. The membrane was then incubated with polyclonal rabbit anti-FP2 (1:5000) and mice anti-FP3 (1:5000) primary antibody for 2 hr followed by washing with 1X PBS for 10 min. The blot was then incubated with 1 hr with secondary HRP-conjugated goat anti-rabbit IgG (Santa Cruz Biotechnology, diluted 1/10,000 dilution in PBS) for FP2 and HRP-conjugated goat anti-mice IgG for FP3 (Santa Cruz Biotechnology, diluted 1/10,000 dilution in PBS) for 2 hr. After incubation, membranes were extensively washed with PBS containing 0.2% Tween-20, and the antigen–antibody complex was developed using DAB tablet (Sigma), and 0.035% of H_2_O_2_.

### Cloning of wild and mutant falcipains

As described earlier pro-enzymes (pro-FP2 and pro-FP3) sequences were amplified from pTOP-pro-FP2 and pTOP-pro-FP3 plasmids, respectively^8,58^. The amplified fragments were digested with BamHI and HindIII (NEB) restriction enzymes for pro-FP2 construct and with BamHI and EcoRI (NEB) restriction enzymes for pro-FP3 construct. The digested fragments were gel extracted and ligated into the expression vector pRSET A (Invitrogen). The sequences of recombinant constructs were confirmed by DNA sequencing. Further, the constructs were transformed into BL21 (DE3) *E*. *coli* expression cells (Qiagen). H205L FP3 mutant was designed using Q5 Site-Directed Mutagenesis Kit (NEB) and primers were generated using the NEBaseChanger^TM^ (http://nebasechanger.neb.com) with H205L FP3 fw: GATAGAATTACTGAACAAAAAAACTAATAGTTTATATAAAAG and H205L FP3 rev: TTTCTGTAATTTTCTGAAAATATTATAAATC. The amplified gene was digested with BamHI and EcoRI restriction enzymes (NEB), ligated into the 6x His-tagged pRSET A expression vector (Invitrogen) and transformed into BL21 (DE3) *E*. *coli* cells (Qiagen). The sequence of the mutant construct was confirmed by DNA sequencing.

### Expression and refolding of the recombinant and mutant enzyme in *E*. *coli*

Recombinant gene expression systems (pro-FP2, pro-FP3, and H205L-FP3) in BL-21 (DE3) *E*. *coli* expression cells were grown in LB media containing ampicillin (100 μg/ml) at 37 °C till optical density reached 0.4–0.6 at λ_600_ followed by induction with 1 mM isopropyl-1-thio-b-D-galactopyranoside (IPTG) at 37 °C for 3 hr. Harvested cells were dissolved in lysis buffer (250 mM NaH_2_P0_4_, 500 mM NaCl and 0.2 mg/ml lysozyme) for 30 min, sonicated for 90 sec pulse rate (BR Biochem Life Sciences) and centrifuged at 15000 rpm for 30 min at RT. The obtained pellet was suspended in solubilization buffer containing 8 M Urea, 20 mM Tris, 250 mM NaCl, pH 8.0 for 30 min followed by centrifugation at 15000 rpm for 30 min at RT and the supernatant was collected for protein purification. Proteins were purified by nickel nitrilotriaceticacid (Ni-NTA) affinity chromatography under denaturing conditions as described earlier^15^. Purification and purity of the recombinant proteins were analysed by SDS-PAGE and quantified by NanoDrop™ 2000/2000c spectrophotometer. Proteins were concentrated using a 10 kDa cut-off filter (Sartorius) to a final concentration of 250 μg/ml^8,58^.

Protein refolding was performed by dialysis method, pro-FP2 was refolded in a Tris-based refolding buffer (100 mM Tris-HCl, 20% glycerol, 250 mM L-arginine, 1 mM EDTA, 1 mM GSH, 0.5 mM GSSG, pH 8.0), while pro-FP3 and its mutant H205L were refolded in 100 mM Tris/HCl, pH 9.0, 1 mM EDTA, 20% sucrose, 250 mM L-arginine, 1 mM GSH and 0.5 mM GSSG) with constant stirring at 4 °C. After 18 hr of incubation, the refolding buffer was exchanged to dialysis buffer (10 mM Tris-HCl; pH 7.5) and again stirred at 4 °C for next 16 hr. Further, dialysed proteins were collected and centrifuged at 15,000 rpm for 15 min at 4 °C.

### Auto-processing assay

300 nM of refolded pro-FP2 and pro-FP3 were incubated in the presence and absence of 10 μM inhibitors NA-01, NA-03, NA-06, and E64 for 10 min followed by activation under acidic conditions of 100 mM sodium acetate pH 5.5, 5 mM DTT and 1 mM EDTA as described previously^15^. Reaction products were resolved by 12% SDS-PAGE and visualized by Coomassie stain.

### Haemoglobin hydrolysis assay

To assess the hydrolysis of Hb, 300 nM of refolded pro-enzymes (pro-FP2 and pro-FP3) and active enzymes FP2, FP3 were incubated with or without 10 μM of compounds NA-01, NA-03, NA-06, and E-64 followed by addition of 8 μg Hb under activation conditions of 100 mM sodium acetate pH 5.5, 5 mM DTT and 1 mM EDTA and incubation at 37 °C for 3 hr as described previously^15^. For wild FP3 and mutant H205L FP3, Hb hydrolysis was performed as described above. Reaction products were resolved using 15% SDS-PAGE and visualized by Coomassie stain.

### Fluorogenic substrate assay

50 nM of refolded pro-enzymes (pro-FP2 and pro-FP3) and active enzymes (FP2 and FP3) were incubated with or without 10 μM of compounds (NA-01, NA-03, NA-06, and E-64) for 10 min followed by addition of 20 μM fluorogenic substrate Z-Leu-Arg-AMC under the acidic condition of 100 mM sodium acetate (pH 5.5), 5 mM DTT for 20 min. The fluorescence (excitation, 355 nm; emission, 460 nm) due to substrate cleavage was continuously measured for 15 min at RT as described earlier^15^. The graph was prepared using GraphPad Prism and the release of AMC was measured after 20 min with fluoro-spectrophotometer (Shimadzu Analytical).

### Circular dichroism analysis of enzymes

Pro-enzymes alone (pro-FP2, pro-FP3), pro-enzymes with inhibitors (NA-01, NA-03) and mutant H205L FP3 were exchanged to 10 mM phosphate buffer, pH 8.0 and concentrated up to 250 μg/ml by 10 kDa cut-off filter (Sartorius). The experiment was performed in a quartz cell of 1 mm path length (Hellma) using chirascan spectropolarimeter (Applied Photo Physics). CD signal was monitored between 190 and 260 nm at 25 °C and each spectrum was obtained by an average of five best scans^15^.

### Biomolecular interaction study

Kinetic measurements of compounds (NA-01& NA-03) were analysed by SPR based biomolecular interaction. The SPR gold chip was activated with a 0.2 M N-ethyl-N′-(dimethylamino-propyl) carbodiimide (EDC) and 0.05 M N-hydroxysuccinimide (NHS) (Sigma) solution. The buffer (10 mM HEPES, 150 mM NaCl, 3 mM EDTA, pH 7.4) constituents were the same as recommended for HBS BIAcore buffer. 500 nM pro-enzymes (pro-FP2 and pro-FP3) were immobilized to the SPR gold chip by amine coupling chemistry at 25 °C with a flow rate of 50 μL/min. Individual compounds (NA-01 & NA-03) in a range of 20 nM to 20 μM were injected to the chip immobilized with enzyme and affinity constant (K_d_) was determined by Autolab ESPRIT instrument^11^.

## Electronic supplementary material

Supplementary Information
