# Supplementary material for: Allosteric Site Inhibitor Disrupting Auto-Processing of Malarial Cysteine Proteases
Source: Sci Rep. 2018 Nov 1;8:16193. doi: 10.1038/s41598-018-34564-8 (PMC6212536; doi:10.1038/s41598-018-34564-8)
Supplement: Supplementary file 1 — Supplementary Information [file 41598_2018_34564_MOESM1_ESM.pdf]

Supplementary Information

Allosteric Site Inhibitor Disrupting Auto-Processing of Malarial Cysteine Proteases

Pant A[1,5], Kumar R[#2], Wani NA[#3], Verma S[1,5], Sharma R[4], Pande V[5], Saxena AK[4], Dixit R[1], Rai R[3], Pandey KC[1,6*].

[1]ICMR-National Institute of Malaria Research (ICMR), Dwarka Sector 8, New Delhi, India

[2]Integrated Science Lab, Umeå University, Umeå, Sweden

[3]Medicinal Chemistry Division, CSIR-Indian Institute of Integrative Medicine, Jammu, India

[4]School of Life Sciences, Jawaharlal Nehru University, New Delhi, India

[5]Department of Biotechnology, Kumaun University, Nainital, Uttarakhand, India

[6]Department of Biochemistry, ICMR-National Institute for Research in Environmental Health, Bhopal, MP - 462001, India

[*]Correspondence and requests for materials should be addressed to K.C.P. (kailash.pandey.nireh@gov.in)

[#] These authors contributed equally to this work.

Contents

1.1. Compounds Synthesis 3

1.1.1. Synthesis of compounds NA-01, NA-02 and NA-03 3

1.1.2. Synthesis of Ac-$\beta^{3,3}$-Ac_6c-$NHNH_2$, NA-04 3

1.1.3. Synthesis of Pyr- $\beta^{3,3}Ac_6c$-OH, NA-05 4

1.1.4. Synthesis of Pyr-Gpn-OH, NA-06 4

2.1. Modeling of whole (pro-mature) domain 4

2.2. Equilibration of the Falcipain 2 model 5

2.2.1. Method 5

2.2.2. Results 5

2.3. Determination of binding site 5

2.3.1. Ensemble structures for docking 5

2.3.2. Ensemble docking by Autodock Vina 6

2.3.4. Rescoring by MM-PBSA 6

2.3.5. Accelerated ligand sampling MD simulations 6

2.3.6. Selection of final complex 6

2.4. Validation by equilibrium MD simulations 6

2.4.1. Interaction network: 7

3. Figures 8

Figure S1. ^{1}H NMR spectrum of NA-01 and NA-02. 8

Figure S2. ^{1}H NMR spectrum of NA-03 and NA-04. 9

Figure S3. ^{1}H NMR spectrum of NA-05 and NA-06. 10

Figure S4: Cavity formation in FP2 and impact of modeled pro- on mature domain conformation during simulations. 11

Figure S5: Workflow schematic to determine Binding site and FP2/NA-03 complex 12

Figure S6: Conformations considered for the ensemble docking. 12

Figure S7: RMSD of FP2 and NA-03 as a function of time for six top complexes 13

Figure S8: Conformational sampling of NA-03 during five equilibrium MD simulations. 14

Figure S9: Interaction of pyrazine and hydrazide groups with N172, R213, K280 and N281. 15

Figure S10. Comparison of active site inhibitor E64 and NA-03 binding site. 16

Figure S11. Multiple Sequence alignment of prodomain from cysteine proteases 17

4. Tables 18

Table S1: List of frame number selected for ensemble docking. 18

Table S2: Rescoring of docked complex using MM/PBSA method. 19

Table S3: Vacuum interaction energy and binding energy between NA-03 and FP2 20

5. References 21

1.1. Compounds Synthesis

All the reagents for chemical synthesis were obtained from Sigma Aldrich and the solvents used in reactions were distilled and dried prior to use. All the chemical reactions were monitored by TLC on 0.25 mm silica gel 60 F254 plates (E. Merck) using UV light as a visualizing agent and ninhydrin as a developing agent. Purification of compounds was carried out by column chromatography using Silica gel 60-120 mesh stationary phase. ^{1}H NMR (with chemical shifts expressed in δ and coupling constants in Hertz) was recorded by Bruker DPX, 400 instruments using $CDCl_3$ as the solvents with TMS as internal standard. High resolution mass spectra (HRMS) were recorded on Agilent Technologies 6540 instrument.

1.1.1. Synthesis of compounds NA-01, NA-02 and NA-03

Scheme 1

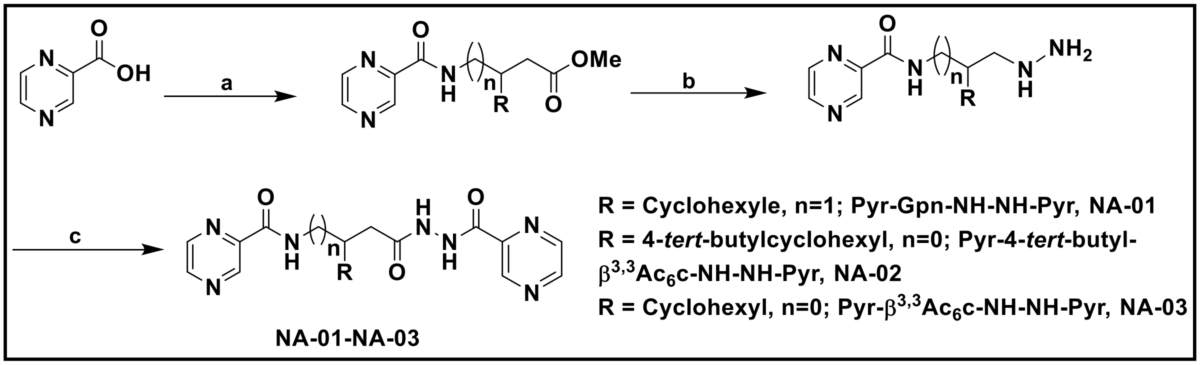

Reagents and conditions: a) Dry DCM, NMM, EDCI.HCl, amino acid ester hydrochloride (Gpn-OMe.HCl, $\beta^{3,3}Ac_6c$-OMe.HCl, *tert*-butyl-$\beta^{3,3}Ac_6c$-OMe.HCl, 0° C to RT, 16 hr; b) Dry MeOH, NH_2NH_2, RT, 12 hr; c) Dry DCM, NMM, EDC, Pyrazine carboxylic acid, 0° C to RT, 24 hr.

Pyrazinecarboxylic acid (0.372g, 3.0mmol) was dissolved in dry dichloromethane (DCM). 400µl of N-methylmorpholine (NMM) was added followed by 1-Ethyl-3-(3-dimethylaminopropyl) carbodiimide hydrochloride EDCI.HCl (0.60 g, 3.0 mmol) and amino acid ester hydrochloride (3.0 mmol) and stirred the reaction mixture for 16 hr. The completion of reaction was monitored by using TLC. Water was added to the reaction mixture and the resulting solution was extracted with ethyl acetate (3×10 ml). The combined organic layer was washed with 2N-HCl (3×10 ml), 1M-Na_2CO_3 (3×10 ml) and brine solution. The organic layer was passed over anhydrous Na_2SO_4 and evaporated under vaccum to give Pyr-Gpn/*tert*-butyl-$\beta^{3,3}Ac_6c/\beta^{3,3}Ac_6c$-OMe. Hydrazine hydrate (485µl, 10 mmol) was added to Pyr-Gpn/*tert*-butyl-$\beta^{3,3}Ac_6c/\beta^{3,3}Ac_6c$-OMe (1.0 mmol) dissolved in dry methanol (1.0ml) and stirred the reaction for 5 hr. The progress of the reaction was monitored by using TLC. The solvent was evaporated completely and the residue left was washed with hexane, acetone and ether to yield the white powder of Pyr-Gpn/*tert*-butyl-$\beta^{3,3}Ac_6c/\beta^{3,3}Ac_6c$-$NHNH_2$.

The pyrazine carboxylic acid (0.124 g, 1.0 mmol) was dissolved in dry DCM and added to 200µl of NMM, EDCI.HCl (0.191g,1.0 mmol) followed by the addition of Pyr-xxx-$NHNH_2$ (0.277 g, 1.0 mmol) and reaction was stirred for 24 h. The progress of the reaction was monitored by using TLC. The reaction was worked up as described in **(1)** to give Pyr-xxx-NH-NH-Pyr, which were purified by column chromatography over silica gel (60-120 mesh) using 5% methanol in chloroform to yield the white powder of Pyr-xxx-NH-NH-Pyr (**NA-01, NA-02 and NA-03**).

N-((1-(2-oxo-2-(2-(pyrazine-2-carbonyl)hydrazinyl)ethyl)cyclohexyl)methyl)pyrazine-2-carboxamide, NA-01:

Yield: 52.5%; ^{1}H NMR (400MHz, $CDCl_3$): δ 10.73 (s, 1H), 9.88 (s, 1H), 9.46 (s, 1H), 9.40 (s, 1H), 8.79 (t, *J* = 1.9 Hz, 2H), 8.57 (d, *J* = 1.3 Hz, 2H), 8.30 (t, 1H), 3.72 (d, *J* = 7.3 Hz, 2H), 2.34 (s, 2H), 1.63 (s, 10H), 1.25 (s, 3H). HRMS-ESI: M_{Cal} = 397.43; M_{Obs} = 398.19 $[M+H]^+$, 420.17 $[M+Na]^+$,436.14[M

N-(4-(tert-butyl)-1-(2-oxo-2-(2-(pyrazine-2-carbonyl)hydrazinyl)ethyl)cyclohexyl)pyrazine-2-carboxamide, NA-02:

Yield: 55.5%; ^{1}H NMR (400 MHz, $CDCl_3$): (Yield: 55.5%**)** δ 9.70 (s, 1H), 9.40 (s, 1H), 9.18 (s, 1H), 8.74 (d, *J* = 2.4, 2H), 8.72 (s, 1H), 8.50 (dd, *J* = 10.1, 1.5 Hz, 2H), 8.03 (s, 1H), 3.15 (s, 2H), 1.32–1.18 (m, 10H), 0.93 – 0.81 (m, 9H). HRMS-ESI: M_{Cal} = 439.52; M_{Obs} = 440.24 $[M+H]^+$, 462.22 $[M+Na]^+$.

N-(1-(2-oxo-2-(2-(pyrazine-2-carbonyl)hydrazinyl)ethyl)cyclohexyl)pyrazine-2-carboxamide, NA-03: Yield: 66.6%; ^{1}H NMR (400MHz,$CDCl_3$): δ 9.86 (s, 1H), 9.38 (s, 1H), 9.20 (d, *J* = 1.2 Hz, 1H), 9.14 (s, 1H), 8.75 (d, *J* = 2.4 Hz, 1H), 8.73 (d, *J* = 2.4 Hz, 1H), 8.53 (d, *J* = 1.6 Hz, 1H), 8.48 (s, 1H), 8.02 (s, 1H), 3.05 (s, 2H), 1.44 – 1.20 (m, 10H). HRMS-ESI: M_{Cal} = 384.40; M_{Obs} = 384.17 $[M+H]^+$, 406.15 $[M+Na]^+$.

1.1.2. Synthesis of Ac-$\beta^{3,3}$-Ac_6c-$NHNH_2$, NA-04

NA-04: N-(1-(2-hydrazinyl-2-oxoethyl)cyclohexyl)acetamide

Scheme 2

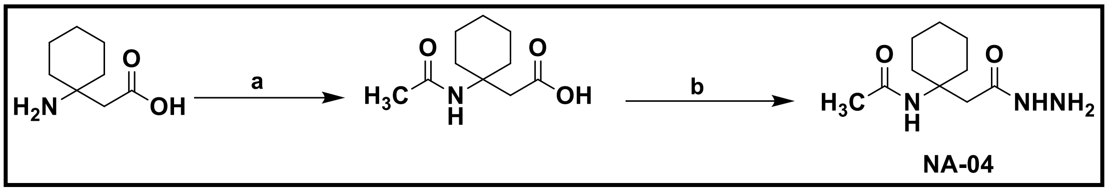

Reagents and conditions: a) Py/(Ac)$_2$O, RT, 3 h; b) Dry DCM, NMM, EDC, $NH_2NH_2.H_2O$

$\beta^{3,3}$Ac$_6$c-OMe (0.855 g, 5.0 mmol) obtained from (1.04 g, 5.0 mmol) of its hydrochloride was dissolved in 1.0ml of pyridine followed by addition of acetic anhydride (0.80 ml, 5.0 mmol) and stirred the reaction mixture for 5 hr. The completion of the reaction was monitored by TLC. The reaction was quenched with 1M-Na_2CO_3 solution followed by acidification with 1N-HCl and extracted with ethyl acetate (3x15.0 ml) and washed with brine solution. The combined organic layer was dried over anhydrous Na_2SO_4 and evaporated under vacuum. The residue was triturated with hexane to give Ac- $\beta^{3,3}$Ac$_6$c-OMe. Hydrazine hydrate (485µl, 10 mmol) was added to Ac- $\beta^{3,3}$-Ac$_6$c-OMe (0.21 g, 1.0 mmol) dissolved in dry methanol (1.0 ml) and stirred the reaction for 5 hr. After completion of the reaction, the solvent was evaporated completely and the residue was washed with hexane, acetone and ether to give Ac-$\beta^{3,3}$-Ac$_6$c-NHNH$_2$ (0.7 g, 75%). **^{1}H NMR (400 MHz, CDCl$_3$):** δ7.24 (s, 1H), 5.46 (s, 1H), 2.69 (s, 2H), 2.15 (d, J = 12.5 Hz, 2H), 1.96 (s, 3H), 1.63–1.24 (m, 10H). **HRMS-ESI:** M_{Cal} = 213.28; M_{Obs} = 214.19 $[M+H]^{+}$.

1.1.3. Synthesis of Pyr-$\beta^{3,3}$Ac$_6$c-OH, NA-05

NA-05: 2-(1-(pyrazine-2-carboxamido)cyclohexyl)acetic acid

The synthesis of Pyr-$\beta^{3,3}$Ac$_6$c-OH was synthesized according to the procedure reported in the literature[1]. Briefly, pyrazine carboxylic acid (3 mmol, 372 mg) was dissolved in dry CH_2Cl_2 and then 200 ml of N-methylmorpholine was added, followed by $\beta^{3,3}$Ac$_6$c-OMe. HCl (3 mmol, 622.5 mg) and EDCI. HCl (3 mmol, 576 mg) at 273 K. The reaction mixture was stirred at room temperature for 12 hr. After completion of the reaction, water was added and the reaction mixture was extracted with CH_2Cl_2 (3×5 ml). The combined organic layer was washed with 2M HCl (2× 5ml), Na_2CO_3 (2×5 ml) and brine solution (2×5 ml). The organic layer was passed over anhydrous Na_2SO_4 and evaporated to give Pyr-^{3,3}Ac$_6$c-OMe.

Pyr-$\beta^{3,3}$Ac$_6$c-OMe (2 mmol, 554 mg) was dissolved in 2 ml of methanol and 1 ml of 2M NaOH, and the reaction mixture was stirred at room temperature for 4 h. Methanol was evaporated and the residue was extracted with diethyl ether (2×5 ml). The aqueous layer was acidified with 2M HCl and extracted with ethyl acetate (3× 5ml). The combined organic layer was washed with brine solution (1×5 ml). The ethyl acetate layer was passed over anhydrous Na_2SO_4 and evaporated to give Pyr-^{3,3}Ac$_6$c-OH (yield: 370 mg, 70.3%).

1.1.4. Synthesis of Pyr-Gpn-OH, NA-06

NA-06: 2-(1-((pyrazine-2-carboxamido)methyl)cyclohexyl)acetic acid

The synthesis of Pyr-Gpn-OH was synthesized according to the procedure reported in the literature[2]. Briefly pyrazine-2-carboxylic acid (3.0 mmol, 0.372 g) was dissolved in dry DCM (3.0 ml) and NMM (0.2 ml) was added, followed by Gpn-OMe. HCl (3.0 mmol, 0.666 g) and EDC. HCl; 3.0 mmol, 0.576 g) under ice-cold conditions. The reaction mixture was stirred at room temperature for 12 hr. After completion of the reaction, water (5.0 ml) was added and the reaction mixture was extracted with DCM (3×5 ml). The combined organic layer was washed successively with 2N-HCl (2×5ml),1-M Na_2CO_3 (2×5 ml) and brine (2×5 ml). The organic layer was passed over anhydrous Na_2SO_4 and evaporated to give Pyr-Gpn-OMe.

Pyr-Gpn-OMe (2.0 mmol, 0.58 g) was dissolved in methanol (2.0 ml) and 2N-NaOH (1 ml), and the reaction mixture stirred at room temperature for 4 hr. The methanol was evaporated off and the residue extracted with diethyl ether (2×5 ml).The aqueous layer was acidified with 2N-HCl and extracted with ethyl acetate (3×5 ml). The combined organic layer was washed with brine (1×5 ml). The ethyl acetate layer was passed over anhydrous Na_2SO_4 and evaporated to give Pyr-Gpn-OH (yield 0.38 g, 69.0%).

2.1. Modeling of whole (pro-mature) domain

The Falcipain 2 (FP2) and Falcipain 3 (FP3) contains two functional domains, residues 165-222 and 261-482, and these two are classified as Inhibitor-I29 and Peptidase-C1 domains, respectively in Pfam database[3,4]. Inhibitor-I29 domain inhibit the enzyme by blocking the active site cavity while Peptidase-C1 acts as protease enzyme. NA-03 inhibits the activation of enzyme, and expected to binds with either Inhibitor-29 domain or at the interface of both domains. However, in the all known crystal structures, coordinates of residues 165-222 are not available[5–7], and therefore, a structure with both domains is required to determine the NA-03 binding site.

To generate the structural model of residues 165-222 by homology modeling method, we searched the template crystal structure in PDB database by querying FP2 (UniprotKB ID: Q9N6S8) and FP3 (UniprotKB ID: Q9NAW4) sequences using NCBI-BLAST online service. Structure of Procathepsin K (PDB:1BY8), Procathepsin L (PDB:1CS8), Procathepsin S (PDB:1COY), Procaricain (PDB:1PCI) were selected as templates to model 155 to 243 and 161 to 249 residues (prodomain) of FP2 and FP3, respectively. Structure of remaining mature FP2 (PDB: 1YVB) and FP3 (PDB: 3BWK) domains were taken as the seed structure, respectively. Subsequently, 50 pro-mature domain models of FP2 and FP3 were generated using Modeller package[8]. These models were scored

using Z-DOPE value, and further, top scorers were evaluated for stereochemical refinement by PROCHECK[9], and finally, best model was selected for subsequent studies.

2.2. Equilibration of the FP2 model

To further validate the above obtained model, we asked whether impact of modelled prodomain on the mature-domain is negligible because mature domain remains similar both in presence and absence of prodomain in FP2 homologs[10,11]. To address this question, we performed equilibrium MD simulations as follows.

2.2.1. Method

At first, starting molecular system for the simulations were prepared as follows. The structure of whole FP2 was obtained by prodomain modelling, and further considered for the simulations. This structure was kept inside a dodecahedron periodic box and subsequently water molecules were added in this box. Further, ions were added to neutralize the system and 0.150 M NaCl was added to simulate physiological ion concentration. For protein and water, this molecular system was further energy minimized by steepest descent method with a force tolerance of 100 kJ $mol^{-1}nm^{-1}$ using GROMACS[12]. Amber99sb-ildn force field[13] and TIP3P[14] parameters were used for minimization and for all further simulations described below.

After the minimization, system was heated from 0 to 300 K with a 2 fs time-step for 100 ps during a constant volume simulation. Next, the density was equilibrated for 1 ns with a 4 fs time-step during NPT simulation. During both the simulations, all heavy atoms of FP2 were restrained at the starting positions with a force constant of 1000 kJ $mol^{-1}nm^{-2}$. In next phase, these restraints were gradually removed in 1 ns NPT simulation with a 4 fs time-step. The temperature and pressure were maintained at 300 K and 1 atm in both NPT simulations using Berendsen temperature and pressure coupling methods[15]. Afterwards, five production MD simulations were performed ranging from 130 to 150 ns. Both temperature and pressure were kept constant at 300 K and 1 atm using v-rescale[16] and modified Parrinello-Rahman[17] methods during the production simulations.

All MD simulations were performed using GROMACS-4.6. During all the above simulations, a 1.4 nm cut-off was used for short range van der Waals and electrostatic interactions. Except during the above heating simulation, this cut-off was applied using Verlet scheme at every 40 steps with 0.005 kJ mol^{-1} ps^{-1} energy drift per atom. For long range electrostatic interactions, PME method was used with a grid spacing of 1.35 A and fourth interpolation order[18]. All bonds were constrained using parallel LINCS algorithm with fourth or sixth order expansion of constraint coupling matrix whenever 2 or 4 fs time-step was used, respectively[19]. All angular bonds containing hydrogen atoms were constrained using virtual sites to allow 4 fs time-steps during the simulations.

2.2.2. Results

To monitor the equilibration, we measured the structural deviations of mature domain during simulations by calculating root mean square deviation (RMSD) with respect to starting crystal structure (Fig. S4c). The obtained RMSD fluctuated at average value of 1.5 °A during these five simulations (Fig. S4c). This observed deviation is considered as quite small in MD simulations because molecules relaxes and fluctuates due to the applied 300K and 1 atm temperature, and pressure, respectively. On considering this quite small structural deviation, the modelled segment has negligible impact on the active domain and, therefore, the obtained MD trajectories were considered for further binding interaction studies.

2.3. Determination of binding site

During the simulations, a cavity was appeared in proximity of an essential hydrophobic cluster (Fig. 1) and at the pro/mature domain interface (Fig. S4b). Additionally, a small cavity was also observed in the prodomain near an essential R185-E221 salt-bridge interaction. Therefore, one of the cavities could be a potential binding site for the designed compounds. We designed a protocol by combining structural and energetic approach to determine the ensemble of structures for docking calculations (Fig. S5).

At first, initial 25 ns of the above obtained five trajectories was discarded by considering as an equilibration phase and further these trajectories were concatenated. The obtained single combined trajectory was used as the input structures for the protocol.

2.3.1. Ensemble structures for docking

In the first part of the protocol, structures at 250 ps time-step were clustered by Jarvis-Patrick method with a threshold root mean square deviation (RMSD) of 1.25 A using *g_cluster* module of Gromacs package. From 49 clusters obtained, 20 were considered for next step because remaining 29 clusters contained less than 5 structures. Each of these 20 clusters contains similar structure, and an energetic approach was used to select a representative structure from each cluster.

In the second part of protocol, folding free energy of structures were calculated from concatenated MD trajectory using FoldX program[20] as follows. Structures from trajectory were extracted at 250 ps time-step and their potential energy was minimized using steepest descent method by GROMACS with a force tolerance of 100 kJ $mol^{-1}nm^{-1}$. These structures were further energy minimized and folding free energy were calculated using FoldX programme[21].

In the next step, folding free energies of above obtained 20 clusters were extracted and a minimum energy structure in each cluster was selected as the representative structure of that cluster (Table S1). At the final step, 20 structures were obtained and its energy minimized configuration was selected for the ensemble docking.

2.3.2. Ensemble docking by Autodock Vina

All the above selected 20 structures were superimposed and this collection of structures form a structure ensemble of the FP2 (Fig. S6). Autodock Vina was used to perform all the docking simulations[22]. Proteins and ligand were prepared by adding polar hydrogen atoms, and assigning Gasteiger atomic charge[23] using AutoDockTools[24]. The search space was defined by placing a box of the 30 Å x 46 Å x 30 Å around the coordinates (centre_x=60, centre_y = 72, centre_z = 40), that covers active site and the interface between the domains (Residues around the box centre: GLN 164, ARG 185, GLU 210, GLU 221, PHE 214, GLN 279, LYS 280, ASN 281, LYS 403, TRP 449, TRP 453, GLY 454). The docking simulations were performed by considering an enhanced exhaustiveness of 32 to maximize the probability of finding global minimum conformation, and 20 binding poses for each ensemble structure were calculated. Overall, 400 (20x20) FP2/NA-03 complexes were obtained after this ensemble docking.

2.3.4. Rescoring by MM-PBSA

To further filter the complexes, we used MM-PBSA method to re-score the obtained NA-03 poses using *g_mmpbsa* tool[25]. This method has been applied previously to rescore the docked poses to remove the false positive poses obtained from the docking.

To perform the rescoring by MM-PBSA, force-field parameters for both FP2 and NA-03 was required. Therefore, Amber99sb-ildn force-field was used for FP2, and its topology-parameter files were prepared using GROMACS.

Partial atomic charges for NA-03 by RESP: For NA-03, partial atomic charges were calculated using RESP method[26] as follows. Its geometry was optimized using HF/6-31G* basis set, and electrostatic surface potential was calculated from the optimized structure using Gaussian 09[27]. Subsequently, atomic charges were calculated by RESP method using *antechamber* module of AmberTools package[28].

Force-field parameters for NA-03: The force-field parameters were taken from general amber force-field (GAFF)[29] using *antechamber* of AmberTools package. The Amber format topology-parameter files of NA-03 were converted to Gromacs format using *acpype* script[30].

After this step, topology and parameter files of FP2 and NA-03 were used and this merged file is further taken as input for rescoring of docked complexes. These force-field parameters were also used for all MD simulations described below.

After the energy calculation by MM-PBSA method, all poses were sorted by the calculated binding energy, and top 19 complexes including two additional complexes with alternate binding sites are listed in Table S2. To select the most likely native binding pose from these 21 poses, we further performed high temperature ligand MD simulations as described below.

2.3.5. Accelerated ligand sampling MD simulations

From the above obtained 21 poses, to select most likely native pose, we used a modified MD simulation protocol to accelerate the sampling of ligand in binding pocket. For these MD simulations, starting molecular systems of 21 complexes were prepared, energy minimized and heated to 300 K as described above for FP2 equilibrium MD simulations. Subsequently, the density of each system was equilibrated for 200 ps with a 2 fs time-step during NPT simulation. During both heating and density equilibration, all heavy atoms of FP2 and NA-03 were restrained at the starting positions with a force constant of 1000 kJ $mol^{-1}nm^{-2}$. In the next step, a 20 ns NPT simulation was performed with 2 fs time-step for each complex and ligand temperature was kept at 500 K to accelerate the ligand sampling while rest of the system was simulated at 300 K. All the simulation settings were kept similar to that of the FP2 equilibrium simulation as per the requirement of 2 fs time-step.

Vacuum interaction energy and MM-PBSA binding energy between FP2 and NA-03: To determine the impact of ligand sampling on interaction, vacuum interaction energy between NA-03 and FP2 for all complexes were calculated from the respective MD trajectory and sorted by their energy values as shown in Table S3. Moreover, average binding energy of top six complexes were calculated using MM-PBSA (**Table S3**).

Impact of ligand sampling on FP2 conformation: To determine the impact of ligand sampling on FP2 structure in top six complexes, root mean square deviation (RMSD) with respect to its starting structure as a function of time was calculated (Fig. S7). According to the binding energy values, strength of NA-03 binding is similar in top two complexes (frame-124: pose-3 and frame-947: pose-3). However, the FP2 RMSD value was increasing throughout the simulations of frame-124: pose-3 while this value oscillated at average of 0.15 nm in case of frame-947: pose-3. These results suggest that structural deviation in FP2 is larger in frame-124: pose-3 than that of the frame-947: pose-3, therefore former complex is not suitable for the enzyme's native structure.

2.3.6. Selection of final complex

From the analysis of above accelerated ligand sampling MD simulations results, N-03/FP2 complex appears to be a highly interacting and least structurally deviating in the frame-947/pose-3 MD trajectory. In this trajectory, both binding energy and FP2 structural deviation remains fluctuated around the average values after 15 ns. A conformation with minimum binding energy at 18.3 ns was extracted and considered for further interaction study.

2.4. Validation by equilibrium MD simulations

The above selected conformation is further prepared, energy minimized, heated and density equilibrated for the equilibrium MD simulation as described above in FP2 equilibrium simulations. Subsequently, five independent equilibrium simulations were

performed for 100 ns (5x100 ns) with 2 fs time step at 300 K temperature and 1 atm pressure. All the simulation settings were kept similar to that of the FP2 equilibrium simulation including the requirement of 2 fs time-step.

2.4.1. Interaction network:

To study the interaction network, a local energy minimum structure was obtained by cooling it to 25 K temperature during simulation. The final structure was used as input for Ligplot+ program[31] to generate interaction network of NA-03 with FP2. To further quantify the interactions, binding energy was calculated by MM-PBSA method using *g_mmpbsa* tool from the combined five MD simulation trajectories[25]. Both methods yielded same residues that are favourable for the NA-03 binding (Fig.7c).

3. Figures

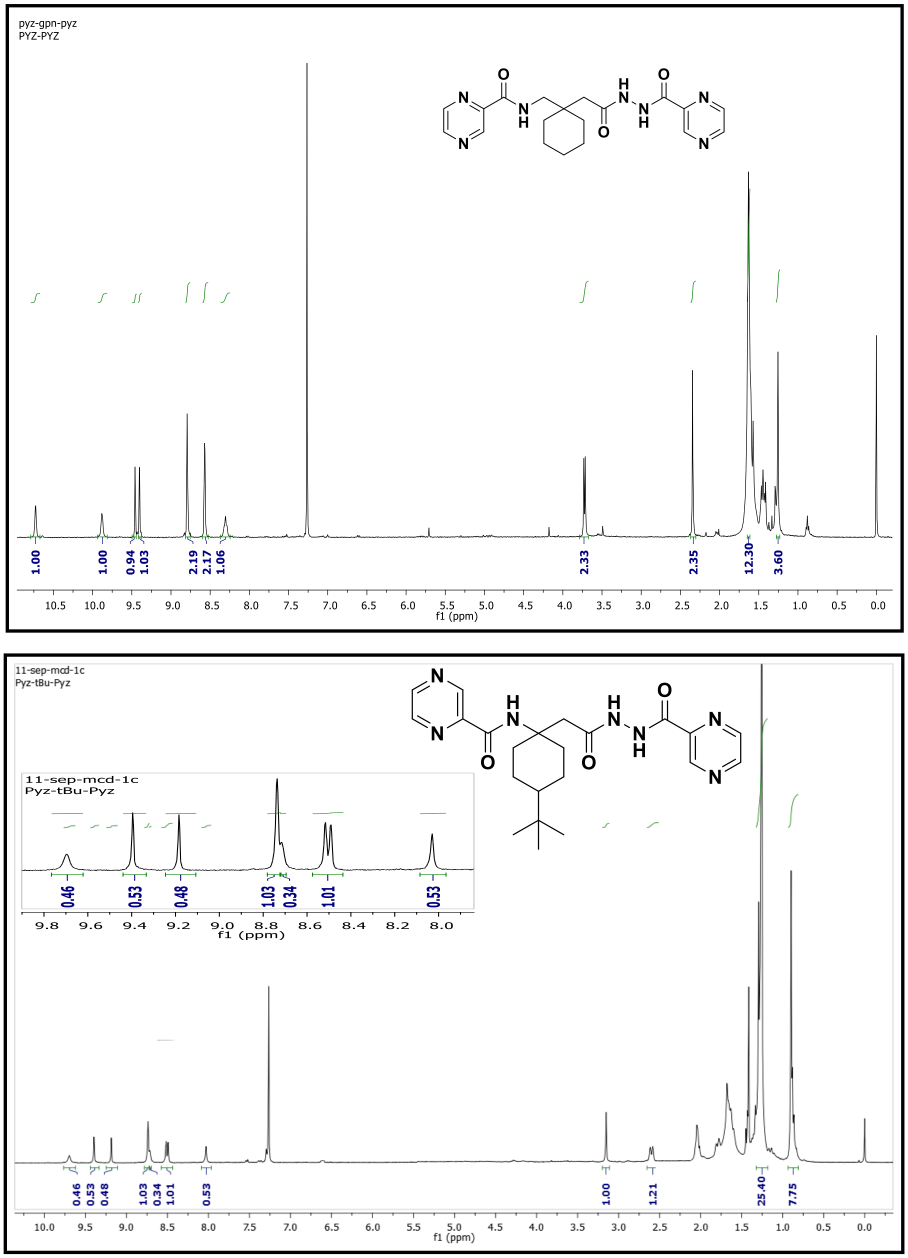

Figure S1. ^{1}H NMR spectrum of NA-01 and NA-02.
400 MHz ^{1}H NMR spectrum of (a) Pyr-Gpn-NH-NH-Pyr, NA-01 in $CDCl_3$ and (b) Pyr-4-tBu-β3,3-Ac_6c-NHNH-Pyr, NA-02 in $CDCl_3$.

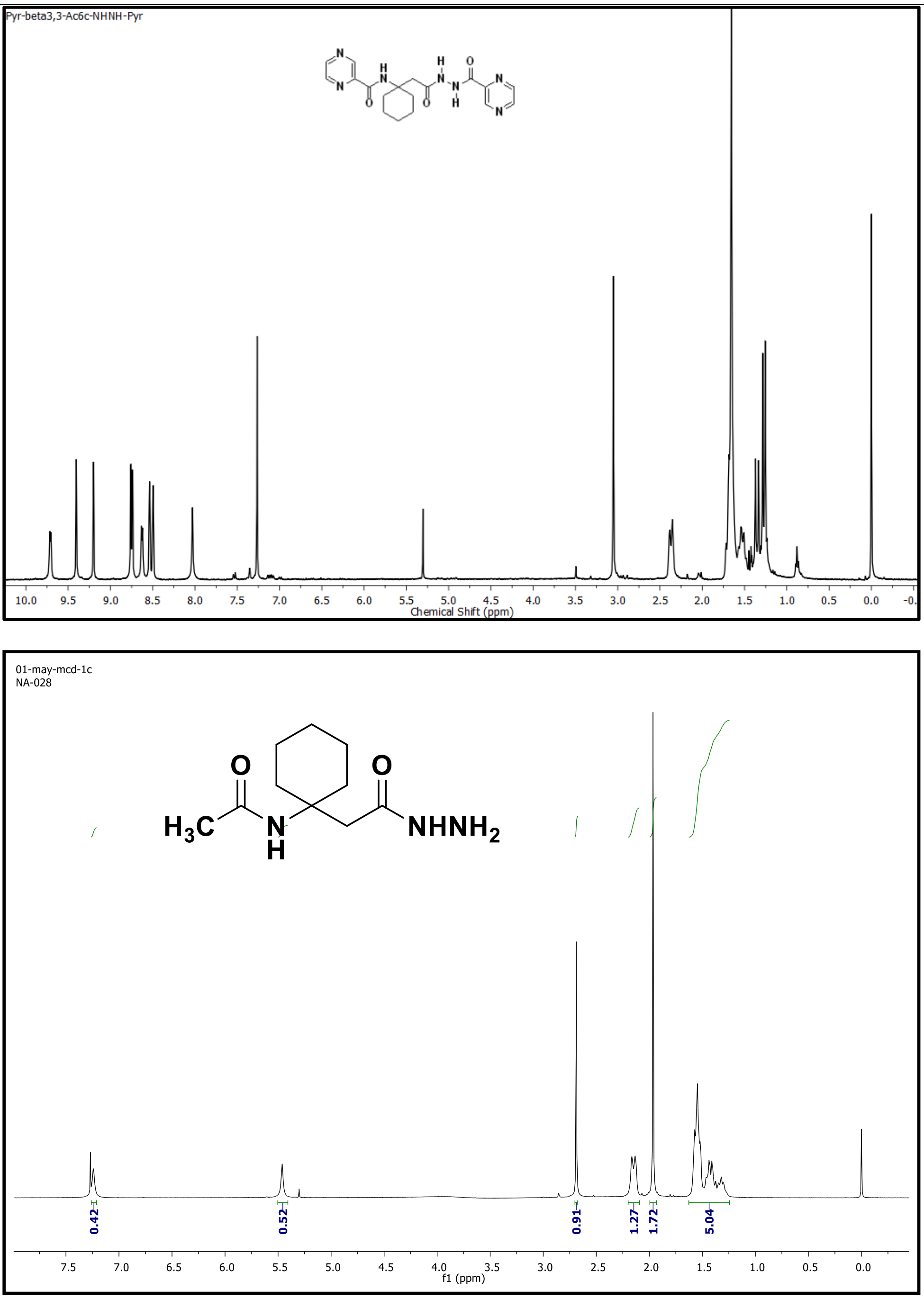

Figure S2. ^{1}H NMR spectrum of NA-03 and NA-04.
400 MHz ^{1}H NMR spectrum of (a) Pyr-$\beta^{3,3}$-Ac$_6$c-NHNH-Pyr, NA-03 and (b) Ac-$\beta^{3,3}$-Ac$_6$c-NHNH2, NA-04 in $CDCl_3$.

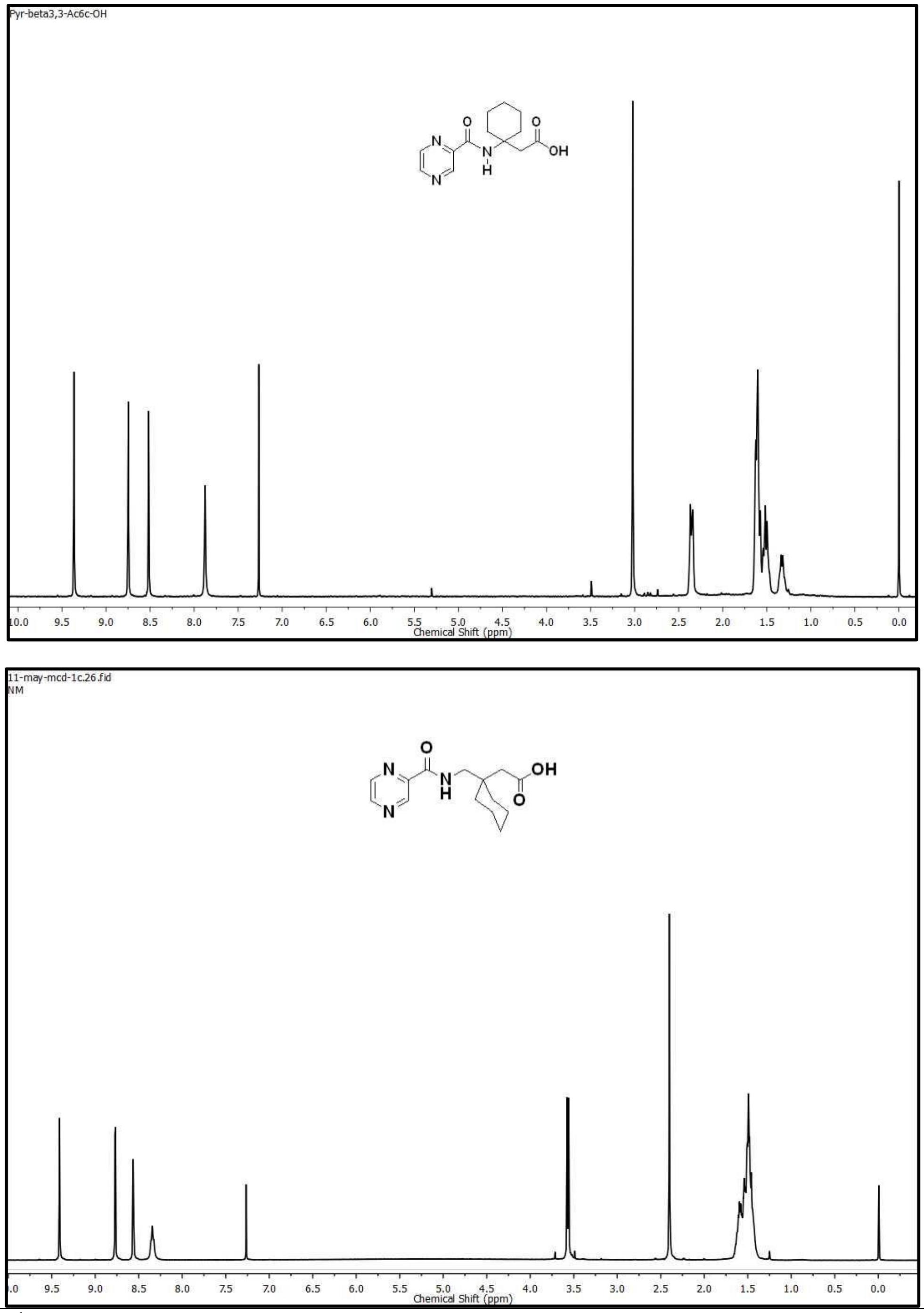

Figure S3. ^{1}H NMR spectrum of NA-05 and NA-06.
400 MHz ^{1}H NMR spectrum of (a) Pyr-β^{3,3}-Ac$_6$c-OH, NA-05 and (b) Pyr-Gpn-OH, NA-06 in $CDCl_3$

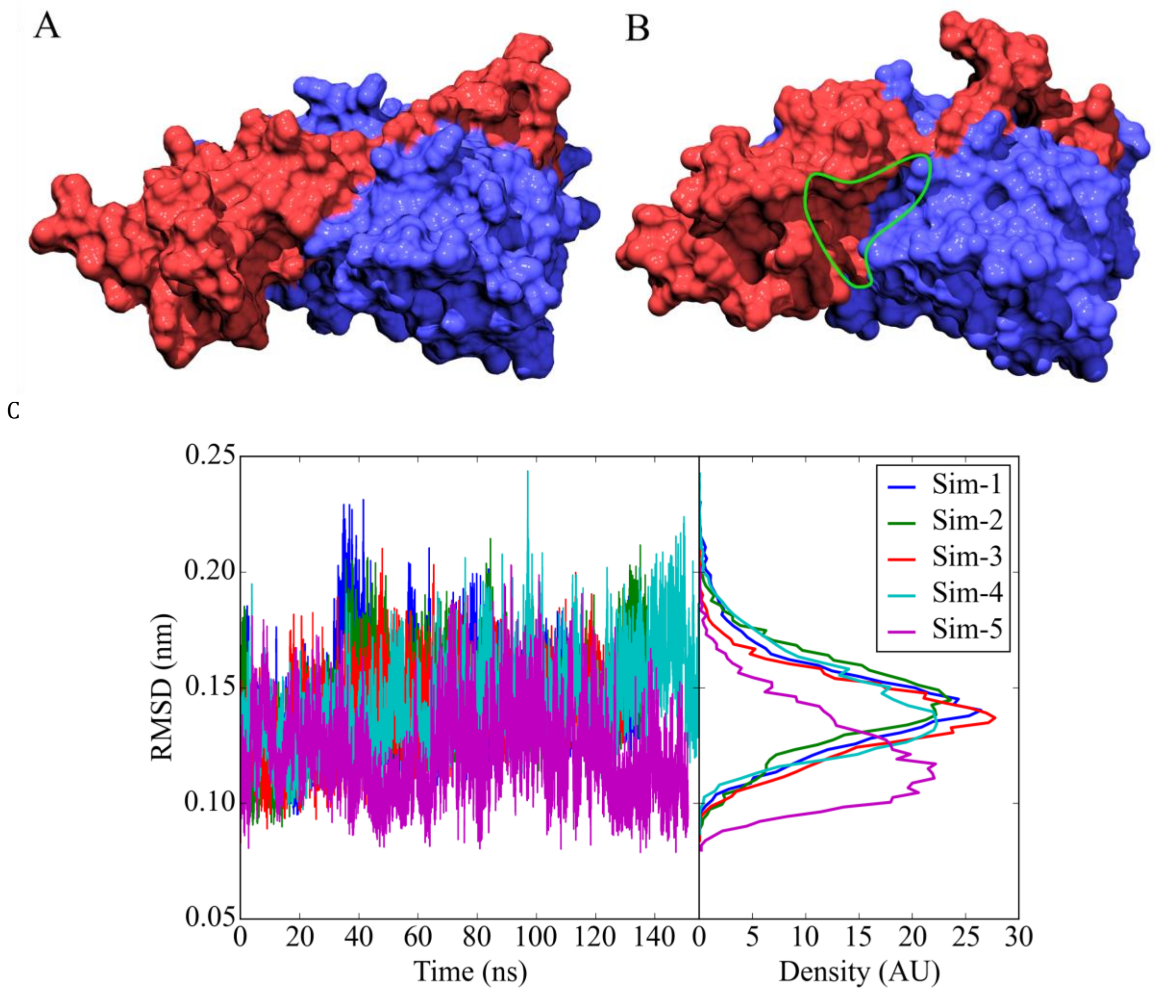

Figure S4. Cavity formation in FP2 and impact of modelled pro- on mature domain conformation during simulations. (**a,b**) Cavity was appeared at pro- (*red*) and mature (*blue*) domain in FP2 during equilibrium MD simulations. (**a**) Cavity was not present at the interface in the starting model and (**b**) cavity appeared (outlined in green) during the MD simulations. (**c**) In left panel, RMSD of C-alpha atoms with reference to the starting structure are shown separately for five trajectories (five colours). In the right panel, frequency of RMSD values are shown for respective trajectories (five colours).

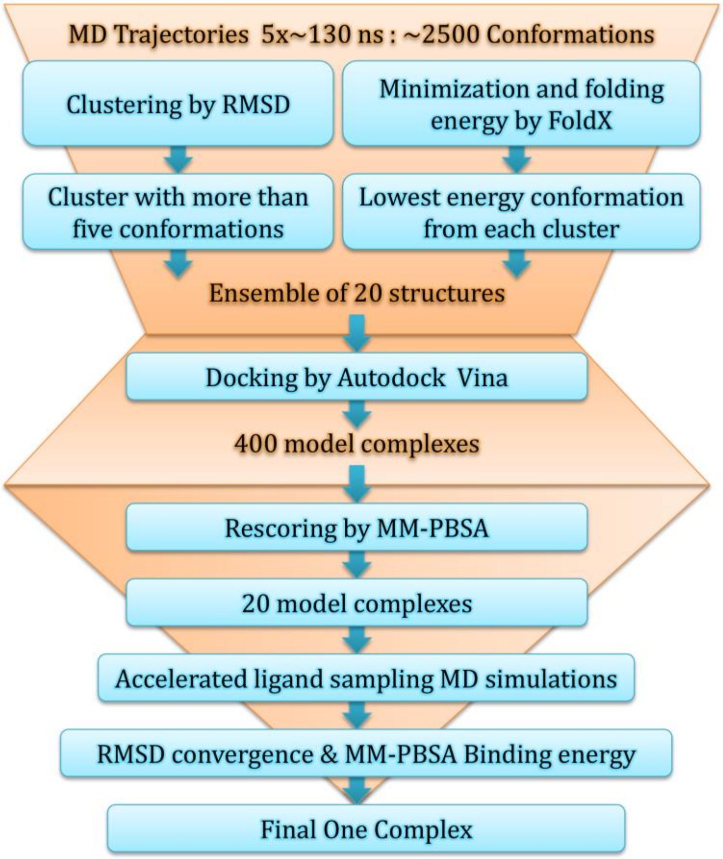

Figure S5. Workflow schematic to determine Binding site and FP2/NA-03 complex by conformational ensemble generation, molecular docking, MM-PBSA rescoring, and accelerated ligand sampling MD simulation.

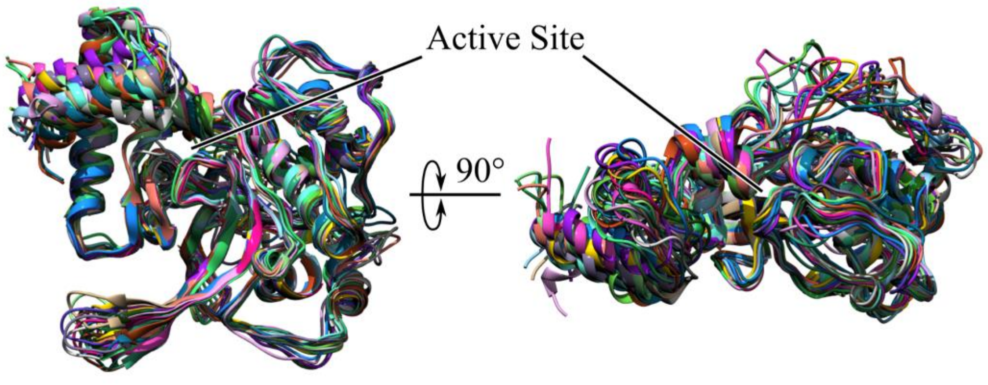

Figure S6. Conformations considered for the ensemble docking.
Shown are the 20 conformations (multiple colours) selected from MD trajectories after employing conformational clustering and free energy calculations using FoldX (http://foldxsuite.crg.eu/).

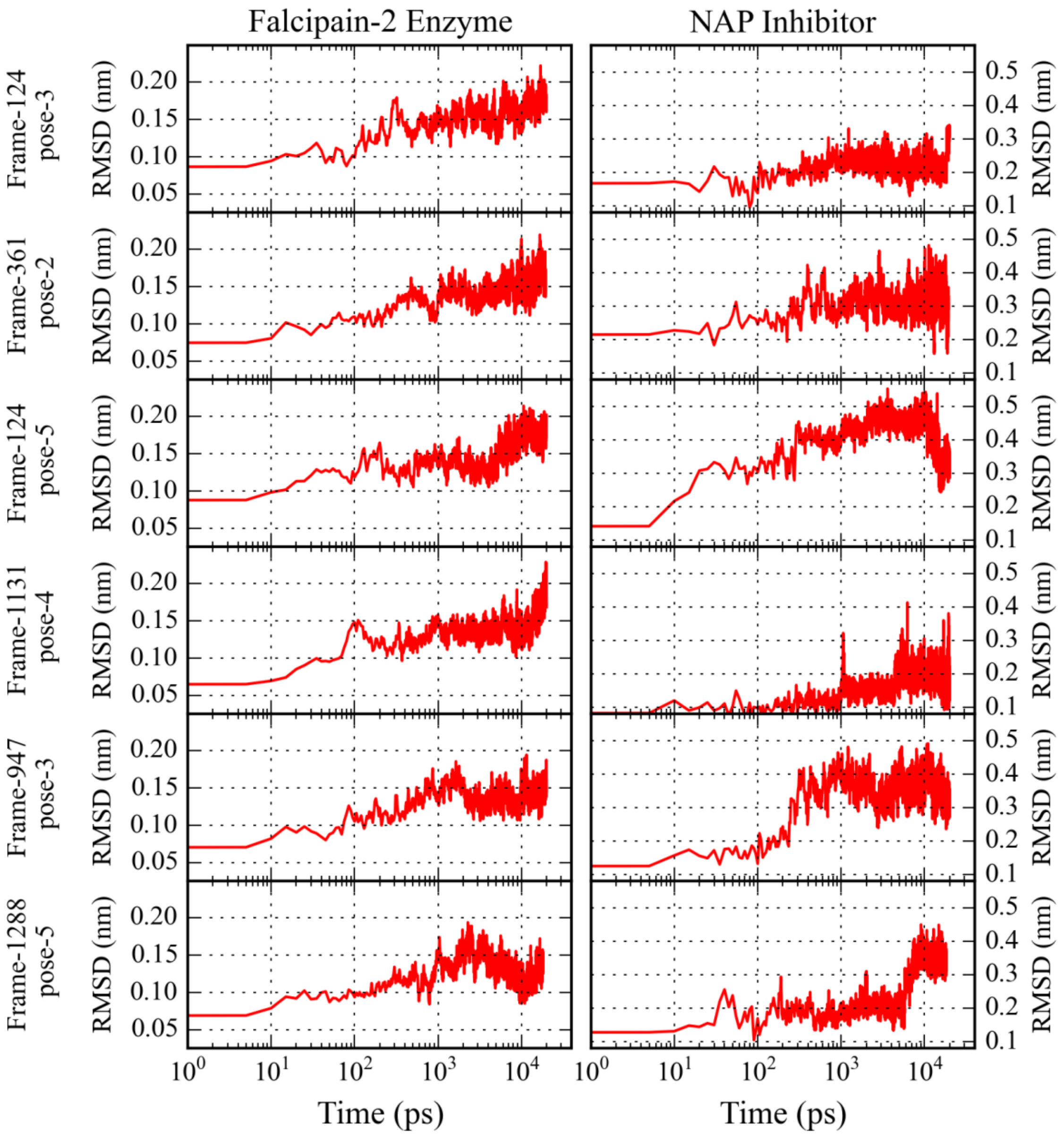

Figure S7. RMSD of FP2 and NA-03 as a function of time for six top complexes (Table. S3) during accelerated ligand MD simulations. Note that time axis is in logarithmic scale.

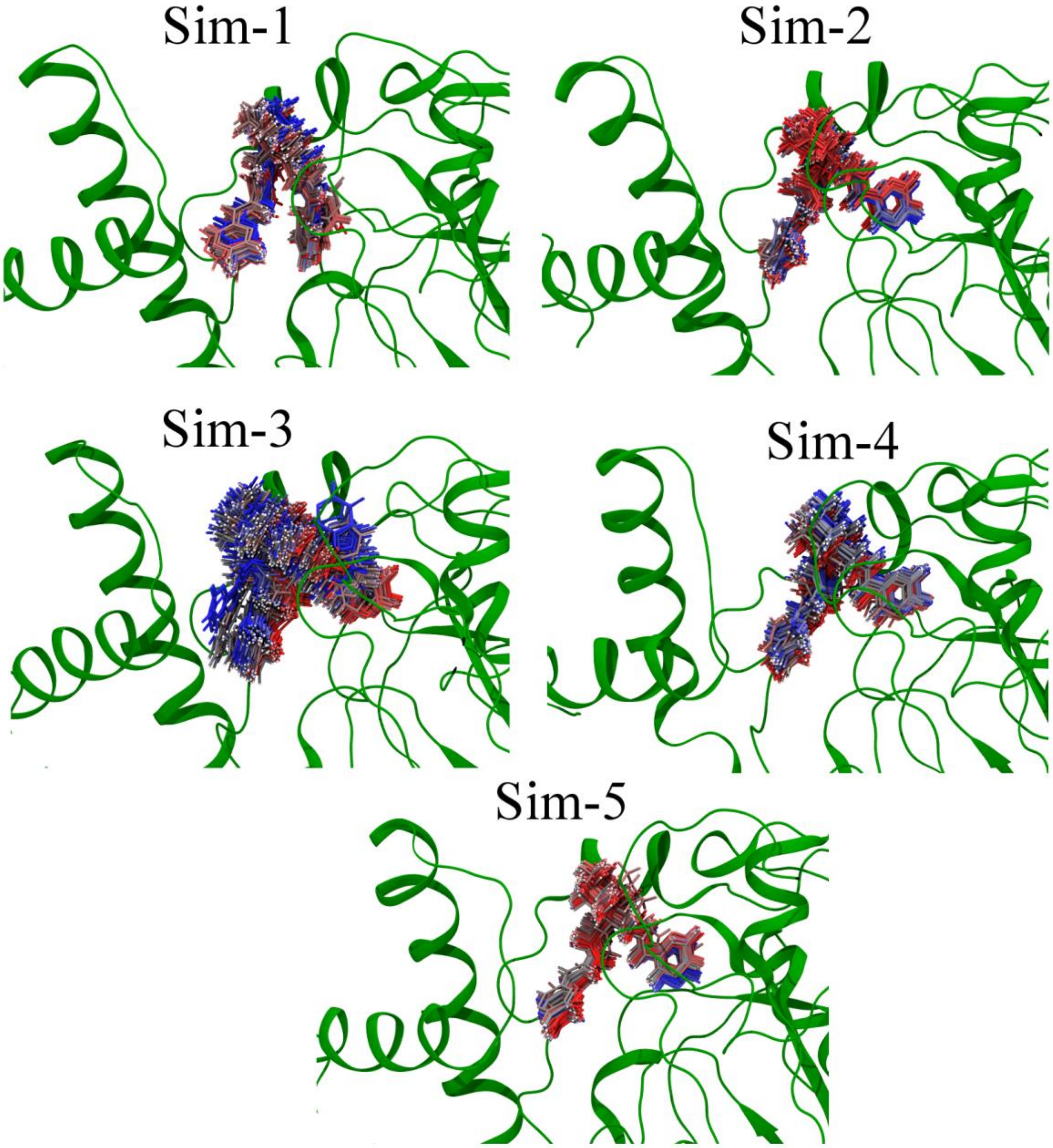

Figure S8. Conformational sampling of NA-03 during five equilibrium MD simulations.
The NA-03 conformations (*red*, *grey* and *blue*) obtained during the five simulations are shown as a function of time in [*red* - *grey* - *blue*] colour-scale. Only one conformation of FP2 (*green*) are shown for clarity.

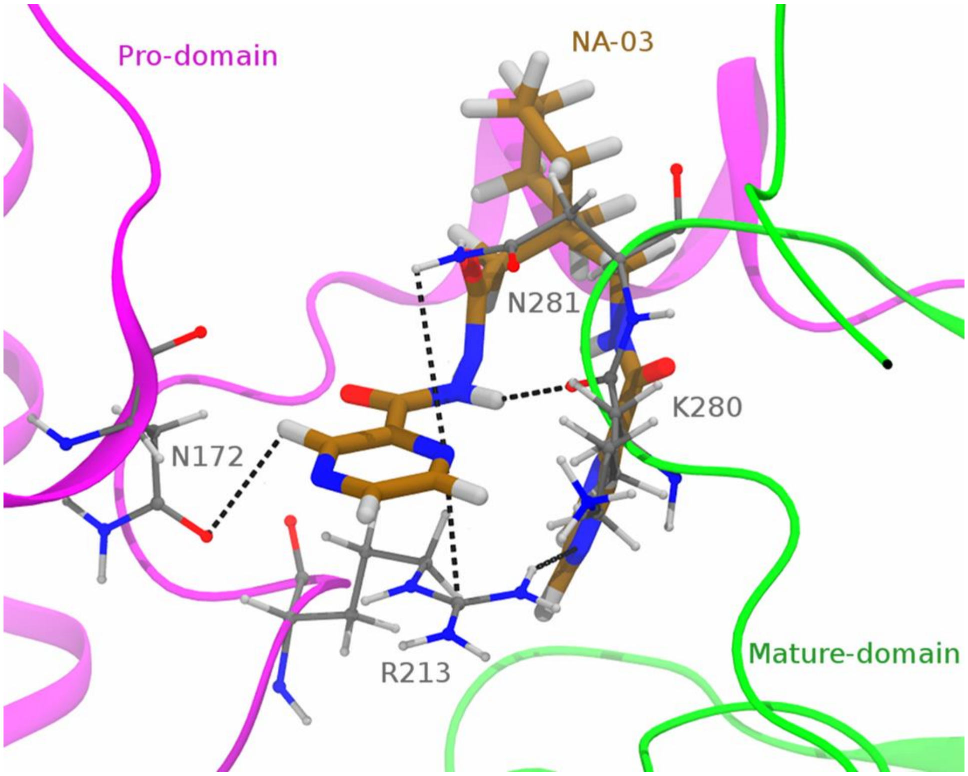

Figure S9. Interaction of pyrazine and hydrazide groups with N172, R213, K280 and N281.
NA-03 pyrazine ring interacts with N172, R213 and N281 while neighbouring hydrazide group interact with K280 backbone.

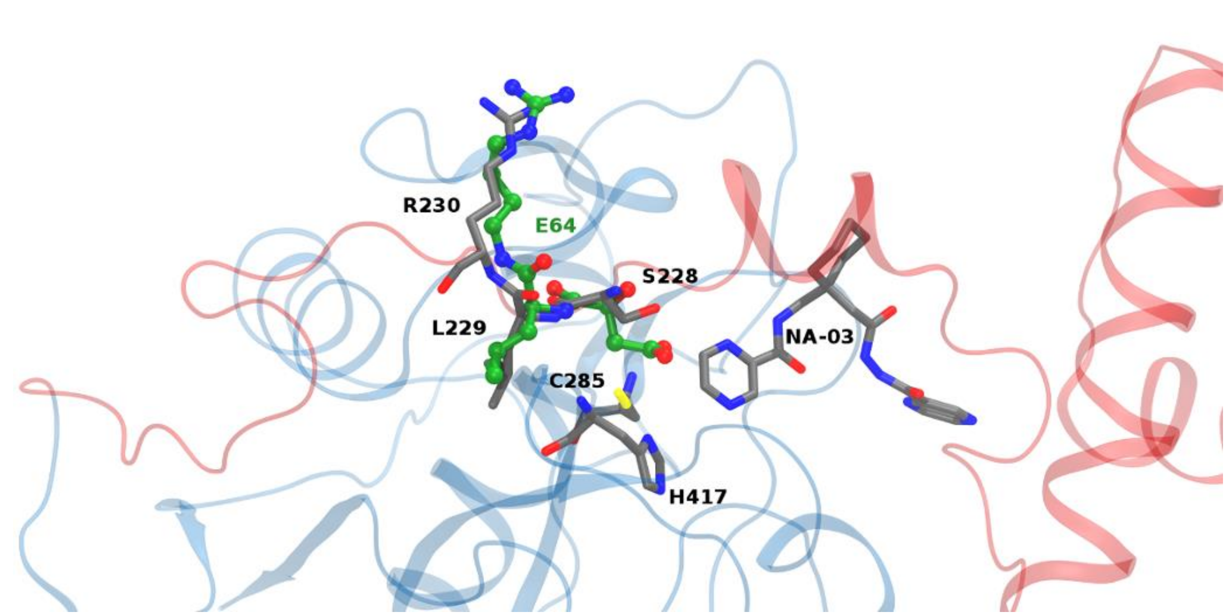

Figure S10. Comparison of active site inhibitor E64 and NA-03 binding site. The E64 (green) binds near to catalytic site (C285 and H417 in grey) of mature-domain (transparent blue cartoon model) and coincides with S228-L229-R230 residues' (grey) position of prodomain (transparent red cartoon model). In contrast, NA-03 binds away from catalytic site and are in contact with prodomain.

Structure of S228-L229-R230 residues and E64 are extremely similar and these three residues blocks the active site cavity in whole enzyme. It is highly unlikely that any ligand will bind to active site in presence of prodomain because it has to displace S228-L229-R230 residues from the active site cavity. Therefore, NA-03 most likely acts as allosteric inhibitor as it is binding away from active site.

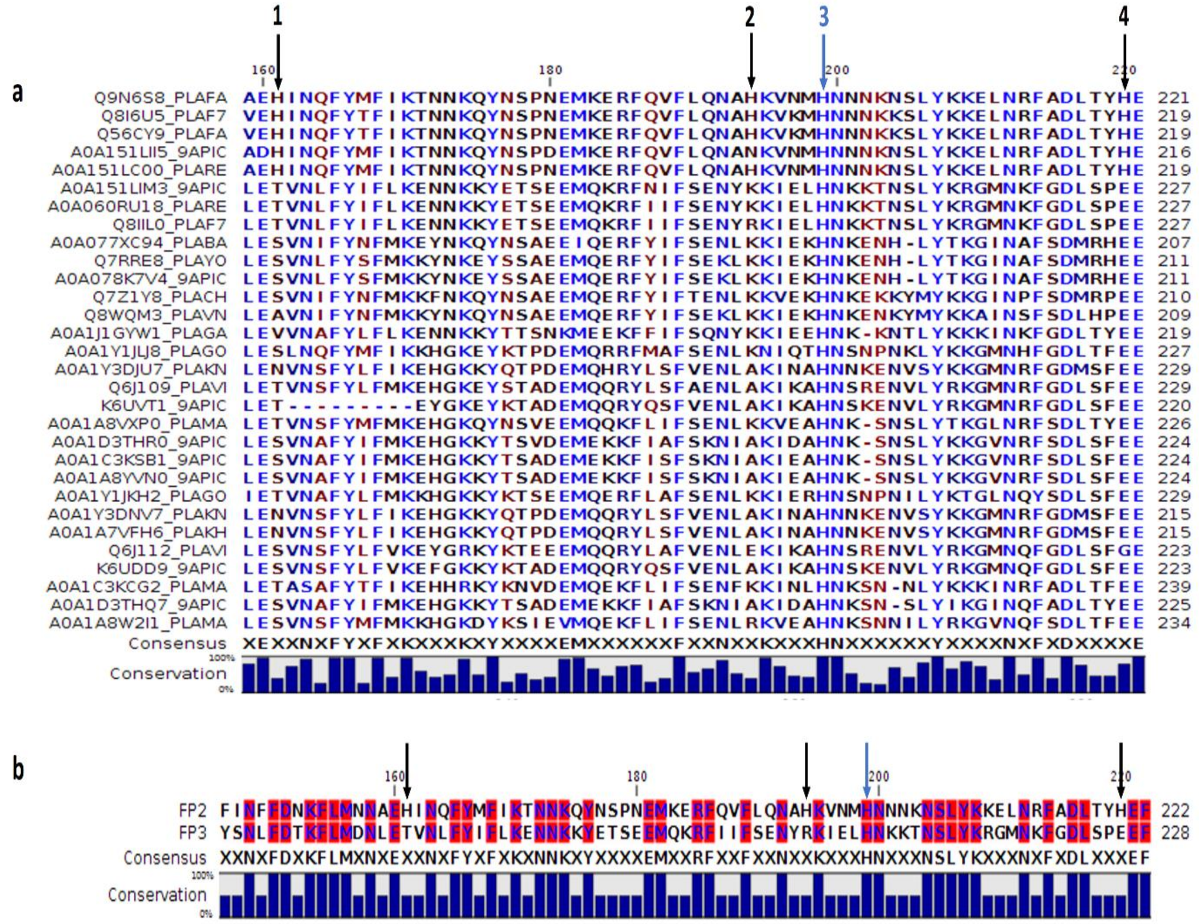

Figure S11. Multiple Sequence alignment of prodomain from cysteine proteases. (a) The sequences of cysteine protease from plasmodium species were aligned by the Clustal Omega server [32,33]. The prodomain region was selected from the alignment for mapping pH sensing histidine residue across the prodomains and coloured according to conservation of residues at each position in the alignment by CLC sequence viewer (https://www.qiagenbioinformatics.com/products/clc-sequence-viewer). Residue numbers are labelled according to the FP2 (Q9N6S8_PLAFA) sequence. The highly conserved residues are in blue letters. The alignment shows that there are four histidine residues positions in FP2 prodomain (H161, H194, H199, H220) and among them H199 is highly conserved across the cysteine protease prodomain of other plasmodium species also. The conserved and pH sensing H199 position is marked by blue arrow and other Histidine position by black arrow. (b) The pairwise alignment of FP2 (Q9N6S8_PLAFA) and FP3 (Q8IIL0_PLAF7) shows the relative residues in FP3 with respect to FP2 histidine residue positions. FP3 have T167, R200, H205 and E226 residues with respect to FP2 histidine residues (H161, H194, H199, and H220).

4. Tables

Table S1: List of frame number selected for ensemble docking. Representative frame from each cluster was selected for the ensemble docking. The representative frame has lowest folding free energy in the respective cluster.

Representative frame number for each cluster	Folding free energy (kJ/mol)
1131	-75
807	-90
1288	-68
9	-77
1770	-88
651	-71
2125	-96
1262	-77
1232	-77
913	-87
947	-84
1620	-74
1365	-91
361	-69
2292	-96
1550	-90
1727	-76
124	-80
1802	-91
639	-68

Table S2: Rescoring of docked complex using MM/PBSA method. The list here shown is sorted according to the MM/PBSA energy values. Only, top 19 out of 400 complexes are listed here. The last two complexes were listed because of very different binding sites. These complexes were taken for the further molecular dynamics simulations.

Frame Number	Pose Number	Energy (kJ/mol)
1232	12	-209
1131	4	-205
1131	13	-188
947	3	-182
1131	8	-179
1131	10	-165
124	12	-161
124	10	-159
361	2	-158
124	5	-157
1232	6	-156
807	18	-156
2292	12	-155
124	3	-152
1620	20	-151
1288	17	-149
1288	5	-146
2292	11	-146
1620	2	-146
807	20	-111
651	13	-101

Table S3: Vacuum interaction energy and binding energy between NA-03 and FP2 during the high temperature ligand MD simulations. The complexes here are sorted in accordance with the total vacuum interaction energy. MM/PBSA was performed to calculate binding energy for top 6 (shown in green and yellow). RMSD of these six complex poses were shown in Fig. S7. The final complex was selected from the simulation highlighted by yellow.

Frame number	Pose number	Vacuum Interaction Energy (kJ/mol)			Binding Energy (kJ/mol)
		van der Waals	Electrostatic	Total	
124	3	-215 ± 14	-97 ± 23	-312 ± 26	-210 ± 5
361	2	-195 ± 15	-100 ± 21	-296 ± 26	-182 ± 5
124	5	-197 ± 17	-81 ± 32	-279 ± 35	-191 ± 6
1131	4	-179 ± 14	-90 ± 22	-270 ± 23	-176 ± 5
947	3	-205 ± 13	-56 ± 16	-262 ± 19	-209 ± 4
1288	5	-174 ± 15	-83 ± 24	-258 ± 27	-169 ± 5
1131	8	-174 ± 10	-74 ± 21	-248 ± 23	Not Done
1131	10	-158 ± 22	-80 ± 33	-238 ± 29	Not Done
1232	6	-177 ± 16	-60 ± 18	-237 ± 26	Not Done
124	10	-169 ± 13	-59 ± 21	-229 ± 25	Not Done
1288	17	-166 ± 13	-62 ± 26	-228 ± 32	Not Done
1232	12	-140 ± 23	-76 ± 18	-217 ± 29	Not Done
124	12	-172 ± 15	-42 ± 15	-214 ± 24	Not Done
2292	11	-107 ± 11	-103 ± 25	-210 ± 29	Not Done
1131	13	-161 ± 17	-44 ± 24	-206 ± 34	Not Done
807	18	-133 ± 33	-62 ± 34	-196 ± 56	Not Done
2292	12	-147 ± 11	-39 ± 16	-187 ± 21	Not Done
1620	20	-129 ± 10	-54 ± 12	-183 ± 16	Not Done
807	20	-118 ± 18	-30 ± 27	-149. ± 30	Not Done
651	13	-100 ± 25	-28 ± 18	-128 ± 37	Not Done
1620	2	-56 ± 32	-21 ± 19	-78 ± 45	Not Done

5. References

1. Wani, N. A., Gupta, V. K., Kant, R., Aravinda, S. & Rai, R. An unusual conformation of gabapentin (Gpn) in Pyr-Gpn-NH-NH-Pyr stabilized by weak inter-actions. *Acta Crystallogr. Sect. C Struct. Chem.* **70,** 776–779 (2014).
2. Wani, N. A., Gupta, V. K., Kant, R., Aravinda, S. & Rai, R. C5 and C7 intramolecular hydrogen bonds stabilize the structure of N-pyrazinoyl-gabapentin (Pyr-Gpn-OH). *Acta Crystallogr. Sect. C Cryst. Struct. Commun.* **69,** 1170–1172 (2013).
3. Finn, R. D. *et al.* The Pfam protein families database: Towards a more sustainable future. *Nucleic Acids Res.* **44,** D279–D285 (2016).
4. Sonnhammer, E. L. L., Eddy, S. R. & Durbin, R. Pfam: A comprehensive database of protein domain families based on seed alignments. *Proteins Struct. Funct. Genet.* **28,** 405–420 (1997).
5. Hansen, G. *et al.* Structural basis for the regulation of cysteine-protease activity by a new class of protease inhibitors in plasmodium. *Structure* **19,** 919–929 (2011).
6. Kerr, I. D. *et al.* Structures of falcipain-2 and falcipain-3 bound to small molecule inhibitors: Implications for substrate specificity. *J. Med. Chem.* **52,** 852–857 (2009).
7. Wang, S. X. *et al.* Structural basis for unique mechanisms of folding and hemoglobin binding by a malarial protease. *Proc. Natl. Acad. Sci. U. S. A.* **103,** 11503–11508 (2006).
8. Eswar, N. *et al.* Comparative protein structure modeling using MODELLER. *Curr Protoc Protein Sci* **Chapter 2,** 2.9.1–2.9.31 (2007).
9. Laskowski, R. A., MacArthur, M. W., Moss, D. S. & Thornton, J. M. PROCHECK: a program to check the stereochemical quality of protein structures. *J. Appl. Crystallogr.* **26,** 283–291 (1993).
10. Li, Z., Kienetz, M., Cherney, M. M., James, M. N. G. & Brömme, D. The crystal and molecular structures of a cathepsin K: chondroitin sulfate complex. *J. Mol. Biol.* **383,** 78–91 (2008).
11. LaLonde, J. M. *et al.* The crystal structure of human procathepsin K. *Biochemistry* **38,** 862–869 (1999).
12. Pronk, S. *et al.* GROMACS 4.5: A high-throughput and highly parallel open source molecular simulation toolkit. *Bioinformatics* **29,** 845–854 (2013).
13. Lindorff-Larsen, K. *et al.* Improved side-chain torsion potentials for the Amber ff99SB protein force field. *Proteins Struct. Funct. Bioinforma.* **78,** 1950–1958 (2010).
14. Jorgensen, W. L., Chandrasekhar, J., Madura, J. D., Impey, R. W. & Klein, M. L. Comparison of simple potential functions for simulating liquid water. *J. Chem. Phys.* **79,** 926–935 (1983).
15. Berendsen, H. J. C., Postma, J. P. M., van Gunsteren, W. F., DiNola, A. & Haak, J. R. Molecular dynamics with coupling to an external bath. *J. Chem. Phys.* **81,** 3684–3690 (1984).
16. Bussi, G., Donadio, D. & Parrinello, M. Canonical sampling through velocity rescaling. *J. Chem. Phys.* **126,** (2007).
17. Nosé, S. & Klein, M. L. Constant pressure molecular dynamics for molecular systems. *Mol. Phys.* **50,** 1055–1076 (1983).
18. Darden, T., York, D. & Pedersen, L. Particle mesh Ewald: An $N \cdot \log(N)$ method for Ewald sums in large systems. *J. Chem. Phys.* **98,** 10089–10092 (1993).
19. Hess, B. P-LINCS: A parallel linear constraint solver for molecular simulation. *J. Chem. Theory Comput.* **4,** 116–122 (2008).
20. Schymkowitz, J. *et al.* The FoldX web server: An online force field. *Nucleic Acids Res.* **33,** (2005).
21. Schymkowitz, J. W. H. *et al.* Prediction of water and metal binding sites and their affinities by using the Fold-X force field. *Proc. Natl. Acad. Sci. U. S. A.* **102,** 10147–10152 (2005).
22. Trott, O. & Olson, A. J. AutoDock Vina: Improving the Speed and Accuracy of Docking with a New Scoring Function, EfficientOptimization, and Multithreading. *J. Comput. Chem.* **31,** 455–61 (2010).
23. Gasteiger, J. & Marsili, M. A new model for calculating atomic charges in molecules. *Tetrahedron Lett.* **19,** 3181–3184 (1978).
24. Morris, G. M. *et al.* Software news and updates AutoDock4 and AutoDockTools4: Automated docking with selective receptor flexibility. *J. Comput. Chem.* **30,** 2785–2791 (2009).
25. Kumari, R., Kumar, R. & Lynn, A. G-mmpbsa -A GROMACS tool for high-throughput MM-PBSA calculations. *J. Chem. Inf. Model.* **54,** 1951–1962 (2014).
26. Bayly, C. I., Cieplak, P., Cornell, W. & Kollman, P. A. A well-behaved electrostatic potential based method using charge restraints for deriving atomic charges: the RESP model. *J. Phys. Chem.* **97,** 10269–10280 (1993).
27. Frisch, M. J. et al. Gaussian 09, Revision A.02. *Gaussian 09, Revision A.02* (2009).
28. D.A. Case, D.S. Cerutti, T.E. Cheatham, III, T.A. Darden, R.E. Duke, T.J. Giese, H. Gohlke, A.W. Goetz, D. Greene, N. Homeyer, S. Izadi, A. Kovalenko, T.S. Lee, S. LeGrand, P. Li, C. Lin, J. Liu, T. Luchko, R. Luo, D. Mermelstein, K.M. Merz, G. Monard, H., D. M. Y. and P. A. K. *Amber 2017*. *University of California, San Francisco* (2017). doi:citeulike-article-id:2734527
29. Wang, J., Wolf, R. M., Caldwell, J. W., Kollman, P. A. & Case, D. A. Development and testing of a general amber force field. *J. Comput. Chem.* **25,** 1157–1174 (2004).
30. Sousa da Silva, A. W. & Vranken, W. F. ACPYPE - AnteChamber PYthon Parser interfacE. *BMC Res. Notes* **5,** 367 (2012).
31. Laskowski, R. A. & Swindells, M. B. LigPlot+: Multiple ligand-protein interaction diagrams for drug discovery. *J. Chem. Inf. Model.* **51,** 2778–2786 (2011).

32. Sievers, F. *et al.* Fast, scalable generation of high quality protein multiple sequence alignments using Clustal Omega. *Mol. Syst. Biol.* **7,** 539 (2011).
33. Goujon, M. *et al.* A new bioinformatics analysis tools framework at EMBL-EBI. *Nucleic Acids Res.* **38,** (2010).
